# Assessing particle count in electron microscopy measurements of nanomaterials to support regulatory guidance

**DOI:** 10.1038/s41598-025-92266-4

**Published:** 2025-04-07

**Authors:** Charlotte Wouters, Vikram Kestens, Eveline Verleysen, Jan Mast

**Affiliations:** 1 Trace Elements and Nanomaterials, Groeselenbergstraat 99, Sciensano, Uccle, 1180 Belgium; 2 European Commission, Joint Research Centre (JRC), Geel, Belgium

**Keywords:** Electron microscopy, Nanomaterial, Precision, Particle count, Regulatory guidance, Particle size, Particle shape, Uncertainty, Nanoscale materials, Nanoparticles, Nanobiotechnology, Nanomedicine, Nanotoxicology, Risk factors

## Abstract

In the European Union, nanomaterials are regulated through different pieces of sectoral legislation. This legislation often requires risk assessments and thus reliable characterization data, for which regulatory guidance generally recommend electron microscopy. The guidance provides best practices for measurements but lacks requirements on how many particles to measure. Using transmission electron microscopy data of nanomaterials, a strategy based on repeated subsampling is proposed to establish, for different particle size and shape measurands, mathematical relationships between particle count and precision, and subsequently to determine the minimum particle count. Our results confirm that the minimum particle count generally depends on the width of the size and shape distributions and that the median of the distribution can be determined with the highest precision compared to other percentiles. Upon combining the precision uncertainty related to particle number with uncertainties from other sources, such as sample preparation, calibration and trueness, we reach an optimal particle count above which additional particle measurements only yield negligible improvements to the combined measurement uncertainty. Our findings offer an experimental approach for determining the minimum particle count to measure particle size and shape by electron microscopy. It enables efficient analyses and facilitates compliance with legislation addressing nanomaterials across various application domains.

**Supplementary Information:**

The online version contains supplementary material available at 10.1038/s41598-025-92266-4.

## Introduction

**Fig. 1:**
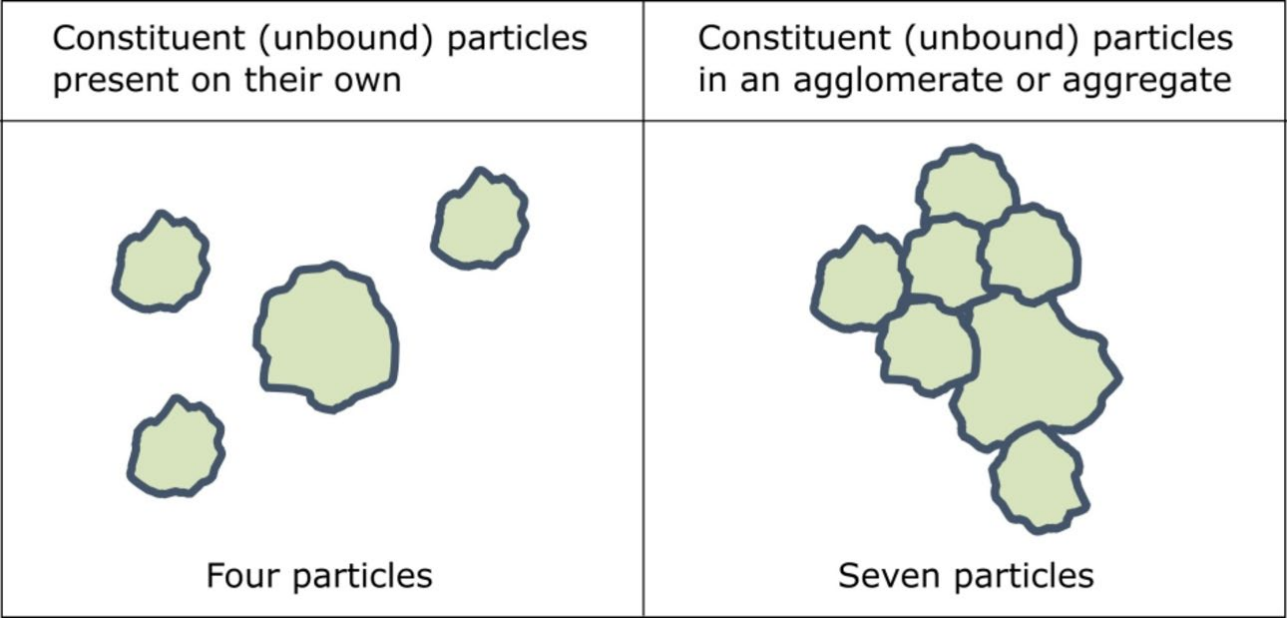
Illustration of the counting of particles with the EC nanomaterial definition (after Bresch et al., 2022^17^).

Nanomaterials may display unique size-dependent physicochemical properties that make them attractive for incorporation in a broad range of applications and products^1^. These products cover, for instance, cosmetics^2^, agro-chemicals^3^, foods^4^, biocides^5^, and medical devices^6^. To minimize the possible health and environmental risks associated with nanomaterials, the European Union (EU) has amended existing regulatory frameworks for such products to specifically address nanomaterials^7–12^, as well as the main legislation for chemicals, REACH^13^. To improve the coherence between these different pieces of legislation, the European Commission (EC) adopted and revised a regulatory definition of “nanomaterial” in 2011 and in 2022, respectively^14,15^. The definitions, which apply to materials that consist of solid particles, differentiate nanomaterials from conventional materials in terms of their particle size distribution and its relative fraction of nanoparticles (i.e. 50% or more of the constituent particles in the number-based size distribution having at least one external dimension in the size range of 1 nm to 100 nm). The EC nanomaterial definitions are more quantitative than other definitions globally^16^. The revised EC nanomaterial definition also requires that the constituent particles that are present on their own and/or in aggregates and agglomerates are counted (Fig. 1).

In supporting the EU’s legislation addressing nanomaterials, specific guidance has been prepared by the European Food Safety Authority (EFSA) and the European Chemicals Agency (ECHA)^18–20^. The implementation of the EC Recommendation on the definition of nanomaterial^14,15^is directly supported by guidance from the EC’s Joint Research Centre (JRC)^21–23^. This guidance promotes a uniform interpretation of the definition across different sectors. It provides clarity by explaining the terminology and concepts used in the nanomaterial definition and offers both a measurement-based decision tree and measurement options to help companies identify nanomaterials effectively^23^. On the other hand, the guidance documents developed by ECHA and EFSA provide comprehensive science-based information to assess the safety of nanoforms, nanomaterials, and materials that contain small particles (i.e. < 500 nm) including nanoparticles taking into account the relevant regulatory specificities. A common element in legislation addressing nanomaterials is the need to reliably measure the size of particles. The JRC, EFSA and ECHA guidance documents recommend established measurement practices and analytical methods for the determination of particle size and particle size distribution.

Within these guidance documents, scanning and transmission electron microscopy (SEM and TEM) are recommended techniques for particle size analysis, since they enable high-resolution imaging and analysis of features at the nanoscale. In addition, they are amongst the very few existing techniques that can effectively measure the external dimensions of constituent particles when present in agglomerates and aggregates (Fig. 1), thus making them key techniques for nanomaterial identification. Compared to routine and high-throughput ensemble methods, such as those based on light scattering and centrifugation measurement principles^24^, SEM and TEM can only observe and measure a small number of particles at a time. To some degree, the statistical significance of results from electron microscopy (EM) analyses can be increased by counting and measuring more particles. This may seem a logical and simple conclusion, but electron microscopes are complex and expensive instruments requiring specialised expertise and, therefore, analyzing more particles come at a significantly higher cost.

Currently, only few guidelines and documentary standards recommend measuring a minimum number of particles to ensure statistically relevant and reliable results. The Test Guideline (TG) no. 125, developed and published by the Organisation for Economic Co-operation and Development (OECD), suggests measuring at least 300 particles for materials with narrow size distributions, i.e. having a geometric standard deviation ≤ 1.5, and at least 700 particles in the case of wider distributions^25^. The documentary standards ISO 21363 and ISO 19749 suggest measuring about 500 particles^26,27^. Both the OECD and ISO methods have derived the minimum particle count, i.e. the minimum number of particles to be measured, from theoretical descriptors associated to log-normal size distributions at a specified confidence level. Depending on the acceptable measurement uncertainty, a lower or higher minimum particle count can be estimated using the methodology described by Masuda^28,29^, as referred to within the guidelines. This study only provides a prediction for the mean of the particle size distribution though, while compliance with regulations is more reliant on the measurement of the median. Not only an accurate determination of the median, but also of multiple percentiles is important as to provide a more complete description of the particle size distribution. An accurate determination of the outer percentiles typically requires more statistics as that of the median, as already shown theoretically by Matsuyama^30^. The theoretical approaches by Masuda and Matsuyama may provide a scientific and valid basis for uniform and spherical particles whose distributions fit well with log-normal functions. However, only little experimental data is available on the correlation and agreement with the main characteristic parameters of particle size distributions obtained on industrially relevant materials^31–33^. In addition, material specific uncertainty data as a function of particle count are lacking for different characteristic size measurands, as well as for other nanomaterial-relevant measurands (e.g. particle shape).

Determining the minimum number of particles to be measured is crucial for ensuring the precision and statistical significance of particle size data from EM. The additional resources required for measuring a greater number of particles may not necessarily result in metrological benefits. We have thus investigated how the particle count correlates with estimates of the precision uncertainty associated to specific percentiles of the particle size and shape distributions of industrially relevant materials, including certified reference materials and representative test materials. Our approach to uncertainty estimation is based on repeated sampling, where subsets of particles are repeatedly drawn from existing TEM particle datasets and subjected to statistical analysis. The results and conclusions of our study provide a basis for updating OECD TG 125 and existing documentary standards, as well as regulatory guidance regarding the minimum particle count to be measured, which can support the implementation of nanomaterial regulatory frameworks in the EU and beyond.

## Materials and methods

### Materials

The analyses were performed for a series of materials (Table 1) covering a variety of particle sizes, shapes and agglomeration states. ERM-FD100^34^ and ERM-FD304^35^ are both colloidal silica reference materials (RM). The former has, however, a higher metrological quality as it comes with a certified particle size value by EM, hence giving it the status of certified reference material (CRM) for electron microscopy applications. On the other hand, the EM particle size value assigned to ERM-FD304 is not certified and it is, therefore, regarded as a non-certified RM. ERM-FD103^36^ consists of titanium dioxide nanorods dispersed in 1-butanol and is certified for different external particle size and shape parameters measured by EM. TiO_2_RM and BaSO_4_RM are titanium dioxide and barium sulfate powder RMs, respectively. Both materials are currently in the process of being characterized and upgraded to CRMs. NM-100 and NM-103^37^ are titanium dioxide powder representative test materials (RTM) and NM-212^38^ is a cerium oxide powder RTM. The RMs/CRMs and the RTMs were obtained from the JRC sites in Geel (Belgium) and in Ispra (Italy), respectively. All these materials have proven stability and homogeneity with respect to the constituent particle size, as illustrated in their accompanying reports. Gold nanorods (AuNRs) dispersed in aqueous medium were purchased from Thermo Fisher Scientific (product number 46945, Karlsruhe, Germany) and is claimed to be stable by the manufacturer under the recommended storage conditions.

**Table 1:** Properties and applied image analysis mode of examined materials.

Material	Material status^1^	Composition	Material type	Particle shape^2^	ParticleSizer analysis mode	Particle size (nm)
ERM-FD100	CRM	SiO_2_	Suspension	Spheroidal	Default	19.4 ± 1.3 ^3^
ERM-FD304	RM	SiO_2_	Suspension	Spheroidal	Default	27.8 ± 1.5 ^4^
ERM-FD103	CRM	TiO_2_	Suspension	Elongated	Irregular watershed + manual intervention	16.1 ± 0.9 ^5^ 54.0 ± 2.4 ^5^
AuNRs	Test material	Au	Suspension	Elongated	Irregular watershed	9–15 ^6^ 60–65 ^6^
TiO_2_RM	RM/candidate CRM	TiO_2_	Powder	Spheroidal	Ellipse fitting	185 ^7^
NM-100	RTM	TiO_2_	Powder	Spheroidal	Ellipse fitting	-
NM-103	RTM	TiO_2_	Powder	Multimodal	Single particle	-
NM-212	RTM	CeO_2_	Powder	Multimodal	Single particle	-
BaSO_4_RM	RM/candidate CRM	BaSO_4_	Powder	Spheroidal	Irregular watershed	25 ^7^

^1^Certified reference material (CRM), non-certified reference material (RM) and representative test material (RTM).

^2^Shape category according to ECHA^20^.

^3^Certified modal value of the ECD PSD.

^4^Indicative modal value of the ECD PSD.

^5^Certified median values of the Fmin and Fmax PSD. Certified values for other measurands are reported in^36^.

^6^Indicative (nominal) values of the particles’ width and length.

^7^Indicative (nominal) median value of the Fmin PSD.

### TEM imaging and analysis

The sample and TEM specimen preparation, TEM imaging and image analysis were performed according to standard operating procedures^39–41^, which were validated specifically to measure the external dimensions of the constituent particles and to determine their number-based particle size/shape distributions (PSDs). Details of the TEM equipment and imaging conditions are given in Supplementary Information A. Image analysis was performed using the ImageJ ParticleSizer plugin software^41^. The applied image analysis mode, as indicated in Table 1, was chosen based on the shape and degree of overlap of the particles in TEM images. The four options of this plugin, ‘Default’, ‘Irregular watershed’, ‘Ellipse fitting’ and ‘Single particle’ are optimized for (i) ellipsoidal particles which are non-aggregated/touching, (ii) irregularly shaped particles which are touching or have a low degree of overlap, (iii) ellipsoidal particles with a low degree of overlap, and (iv) ellipsoidal or irregularly shaped particles with a high degree of overlap, respectively. For material ERM-FD103, which displays many agglomerated nanorods, ‘Single particle’ mode would be the advised mode. Given that single particles were largely absent in the images, the ‘Irregular watershed’ mode was used, and the analysis was manually refined by removing any incorrectly detected particles. PSDs were constructed for four size measurands: the minimum (Fmin) and maximum (Fmax) Feret diameters^42^, the maximum inscribed circular diameter (MICD) and the area-equivalent circular diameter (ECD); and a shape measurand: the aspect ratio (AR), defined as the ratio of Fmax to Fmin. For materials where ‘Ellipse fitting’ was used as ParticleSizer mode, the length of the short and long ellipse axes were used as estimates of Fmin and Fmax of the particles, respectively. The normalized interquartile range of the PSD, IQR%, defined as the ratio of the interquartile range (IQR) to the median of the PSD multiplied by a factor of 100, is calculated as a normalized measure for the width of the PSD. Datasets used for the estimation of the total measurement uncertainty were acquired by repeated measurements on the same day and on different days, according to the experimental design described in Verleysen et al.^32^. Representative TEM images and histograms of the Fmin are available in Supplementary Information B.

### Estimation of uncertainty due to particle count

For each material, the largest available dataset (*N*_tot_) obtained during a single measurement as part of the validation study was selected and used to investigate the relation between the particle count (*N*) and the related precision of the characteristic values of the PSD. A random particle subset of *N* particles was drawn from the full set of particles with replacement. The percentile values D10, D25, D50, D75, and D90 of the number-based distribution of the measurand were calculated from the subset. These two steps were repeated 500 times (as justified in Supplementary Information C), resulting in five sets of 500 percentile values (*D*_*i*_), for each of which the mean$\:\:\stackrel{-}{D}$ and the standard deviation *σ* were determined:

1 $$\sigma=\sqrt{\frac{{{\sum\:}_{i=1}^{500}({D}_{i}-\stackrel{-}{D})}^{2}}{499}}$$

This process was repeated for increasing values of *N* between 10 and *N*_tot_. Based on *σ*, the uncertainty purely associated to the number of measured particles (*U*_N_) for a specific percentile and measurand with a confidence level of 95% (defined by the coverage factor *k*) was determined for each value of *N*:

2 $${U}_{N}=2\frac{\sigma}{\stackrel{-}{D}}\quad\left(k=2\right)$$

The UnivariateSpline function in Python was used to fit a smooth spline function to the data, linking *σ* and *U*_N_ to *N*, allowing an estimation of the minimum particle count (*N*_m_) required to reach a specific value of *U*_N_. Since the calculation of *U*_N_ is based on subsets of particles measured under exactly the same conditions, it does not take into account variations due to sample preparation, EM specimen preparation, imaging or analysis.

### Total expanded measurement uncertainty as function of *N*

To evaluate the contribution of *U*_N_ to the full uncertainty budget, including variations introduced by sample preparation, EM specimen preparation, particle count, imaging and analysis, the total expanded uncertainties (*U*_cx,_ *k* = 2) on the percentiles of the Fmin PSD were calculated as a function of *N*, for selected materials. These calculations are based on validation datasets from TEM measurements obtained on the same day and on different days, to include both within-day variations and between-day variations which are combined into the uncertainty associated to intermediate precision (*u*_IP_). Subsets of size *N* were randomly drawn from each of the validation datasets. Based on these subsets, *U*_cx_ was calculated as the square-root of the sum of the squared standard uncertainties related to intermediate precision (*u*_IP_), trueness (*u*_tr_) and calibration (*u*_cal_), following the methodology as described in Verleysen et al. This process was repeated 500 times for each *N* to reach averaged values of *u*_IP_, *u*_tr_ and *U*_cx_ (*u*_cal_ is constant) over 500 different subsampling simulations. The log transformed data of *U*_cx_ versus *N* are fitted in Python by a piecewise linear function.

## Results and discussion

### Particle size and shape

Table 2 summarizes the median values (D50) and IQR% of the PSDs for the different size and shape measurands obtained from TEM measurements, considering the largest dataset. The median Fmin values, and in selected cases the modal ECD values, with associated expanded uncertainties, *U*_cx_, based on intra-laboratory validation studies, are reported along. The *U*_cx_ values were calculated for a fixed particle count per dataset, which is why they might differ slightly from the results reported by Verleysen et al.^32^, where each validation dataset consisted of the maximum amount of particles obtained from 10 different TEM images. A comparison of the validation data with the certified and indicative values given in Table 1, demonstrates the validity and accuracy of the TEM measurement method, at least for the colloidal CRMs. The trueness of the TEM method could not be assessed for the more complex materials due to the lack of certified reference materials and, as a result, the presence of significant biases resulting from sampling and/or image analysis cannot be completely excluded.

**Table 2:** Particle size and shape statistics of the TEM datasets used for the calculation of *U*_*N*_.

Material	*N*_tot_	Fmin		Fmax		MICD		ECD		AR		Particle size and U_cx_^1^ (nm) from validation study
		D50 (nm)	IQR%	D50 (nm)	IQR%	D50 (nm)	IQR%	D50 (nm)	IQR%	D50	IQR%	
ERM-FD100	11,653	16.7	27	20.1	23	16.9	27	18.5	25	1.1	15	17.4 ± 1.7 ^2^ (19.2 ± 1.9)^3^
ERM-FD304	17,147	23.3	20	26.3	17	23.2	20	24.9	18	1.07	8	22.9 ± 1.6 ^2^ (25.5 ± 1.8)^3^
ERM-FD103	661	15.9	18	48.3	30	15.6	18	28.5	21	2.98	31	16.0 ± 1.8 ^2^
AuNRs	4119	15.8	15	46.8	16	16.3	13	27.4	12	2.96	17	15.8 ± 2.0 ^2^
TiO_2_RM	11,347	169	46	223	47	169	46	194	45	1.27	19	182 ± 20 ^2^
NM-100	3248	103	45	124	52	103	45	113	48	1.91	9	100 ± 8.5 ^2^
NM-103	1119	18.1	52	30.6	72	17.0	45	24	59	1.59	44	18.2 ± 1.8 ^2^
NM-212	1100	16.9	55	25.2	69	16.0	46	20.6	55	1.33	43	15.6 ± 3.4 ^2^
BaSO_4_RM	17,523	26.4	68	35.8	68	25.9	66	30.5	67	1.25	25	27.0 ± 3.2 ^2^

^1^Total expanded measurement uncertainty (95% confidence level).

^2^Median (D50) value of the Fmin PSD.

^3^Modal value of the ECD PSD.

### Relation between *U*_N_ and IQR% for D50

**Fig. 2:**
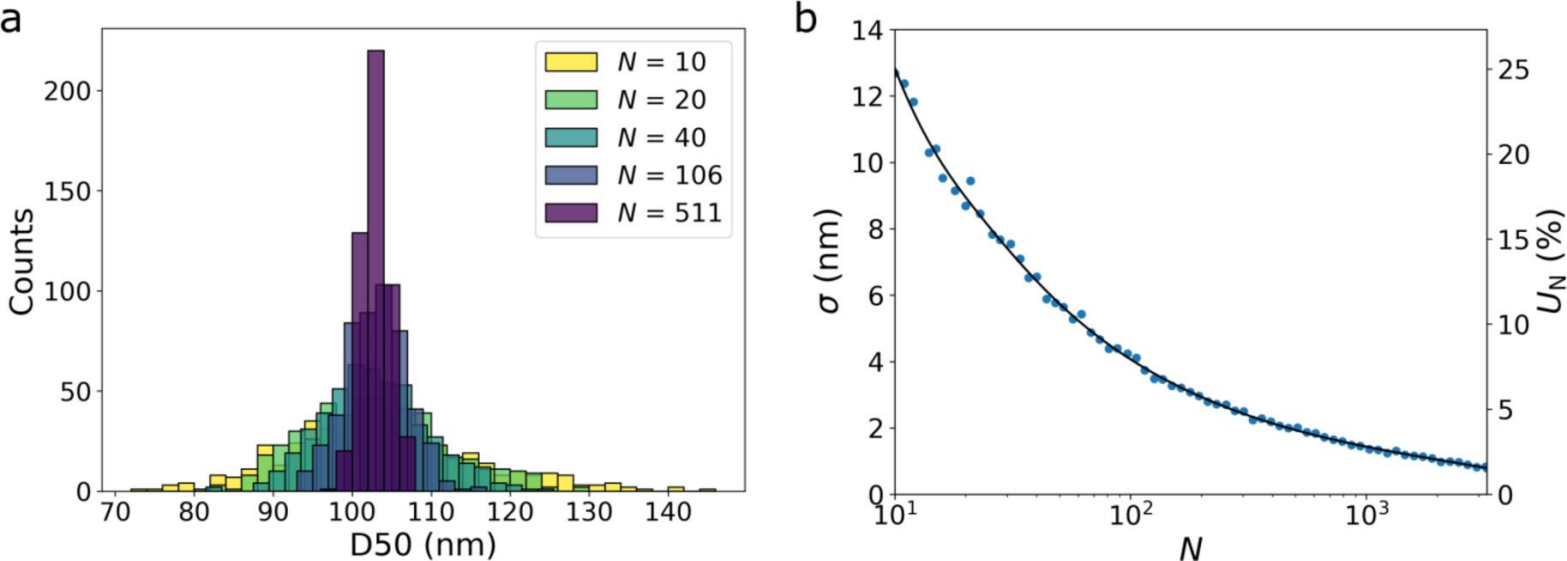
Histograms of Fmin D50 values obtained from 500 particle subsets of *N* particles of material NM-100 (**a**). The standard deviation (left axis) and uncertainty at 95% confidence level (right axis) on the measurement of D50 as a function of *N* with a spline fitting function (**b**).

The approach to estimate *U*_N_ on the PSD percentiles as a function of particle count, based on experimental TEM data (Fmin, D50), is illustrated for material NM-100 in Fig. 2. Comparison of the histograms shows that the spread of the D50 values diminishes when *N* increases (Fig. 2a). Figure 2b confirms the decrease of *σ* and *U*_N_ when *N* increases. From the fitting function one can obtain straightforwardly the minimum particle count, i.e. at least 62 and 267 particles need to be measured to reach a *U*_N_ value of 10% and 5%, respectively.

**Fig. 3:**
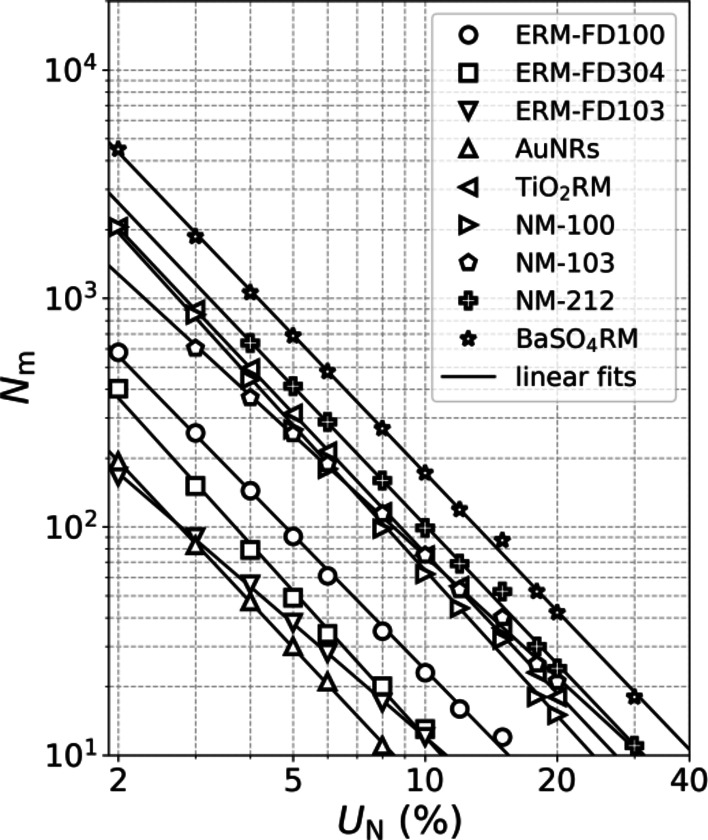
The minimum particle count *N*_m_ for measuring the median Fmin as a function of *U*_*N*_, with linear log-log fits to the data.

Plotting the relation between *N* and *U*_N_ on a log-log plot, results in a linear relationship for all materials, as illustrated in case of D50 of Fmin in Fig. 3. Similar linear relations between the log-transformed data of *N* and *U*_N_ have been established for all other percentiles and measurands, and are available in Supplementary Information D. The curves are ordered by increasing value of IQR% towards increasing values of *N* allowing an estimation of *N*_m_ as a function of *U*_N_ when IQR% of the measurand distribution is known. This estimation can be done *ex post*, as a part of the quality control of the measurement, or *ex ante*, based on partial measurement results or available data (see Supplementary Information E). The linear log-log dependency between *N*_m_ and *U*_N _was already established by Masuda and Gotoh^28^(for the mean diameter) and Matsuyama^30^ based on theoretical derivations and simulations for perfectly lognormal particle size distributions. Our findings show that this qualitative relationship can be extended to real world materials having size distributions deviating from lognormality, independently from particle size, shape, and material polydispersity. The log-log relationship between *U*_N_ and *N*_m_ implies that, at some point, measuring more particles will only lead to a marginal decrease in *U*_N._ In Supplementary Information D, our data are compared with the theoretical derivation by Matsuyama with a discussion of small inconsistencies due to limitations in the experimental uncertainty at high *N* and the fact that the experimental data exhibit a histogram that deviates from a smooth and perfectly distributed shape.

**Fig. 4:**
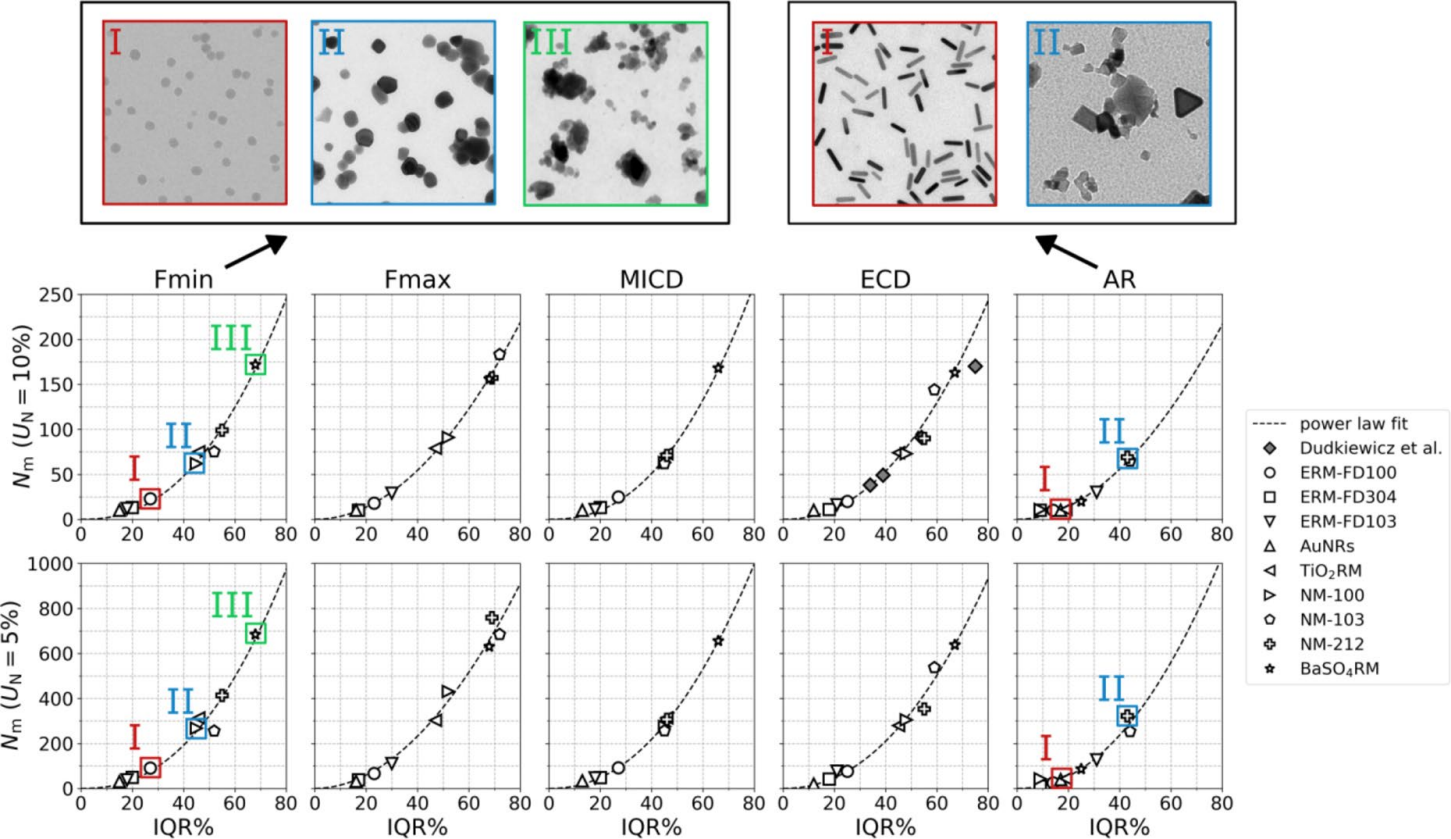
The minimum particle count to reach *U*_*N*_ of 10% and 5% at a 95% confidence level (*k* = 2) of the measurement of the median Fmin, Fmax, MICD, ECD and AR as a function of IQR%, with power law fit to the data. For comparison, data for *N*_m_ (*U*_N_ = 10%) for ECD by Dudkiewicz et al.^31^ have been added. The top shows illustrative TEM images of the material categories I, II and III representing different IQR% ranges for size (Fmin) and shape (AR).

In Fig. 4, power-law functional relationships between *N*_m_ (*U*_N_ = 10%) and *N*_m_ (*U*_N_ = 5%) and IQR% are depicted for the different materials and measurands. The broader the PSD, the more polydisperse is the material and the more particles need to be measured to achieve a given *U*_N_. For AuNRs and BaSO_4_RM, which have the most narrow (IQR%_Fmin_ = 15) and broadest (IQR%_Fmin_ = 68) PSD, respectively, and considering *U*_N_ = 10%, it can be found that as few as 10 and 180 particles need to be counted and measured. The minimum particle count increases to 42 and 896, respectively, for *U*_N_ = 5%. The obtained relationship between IQR% and *N*_m_ has been validated previously by Dudkiewicz et al., who adopted a similar repeated sampling procedure and reported comparable functional relationships (Fig. 4) based on SEM/TEM data for synthetic amorphous silica and Ag nanoparticles in pristine form and applied in food matrices.

Based on the obtained power law behaviour between *N*_m_ and IQR% and the distribution of materials on the curves, three different material categories can be distinguished, each covering a specific range of IQR% and corresponding range of *N*_m_. Note that while this grouping emerges naturally for the selected materials, other materials might fall anywhere on the curve, rendering this categorization as arbitrary. Nevertheless, the provided categories and corresponding example images (Fig. 4) can be useful to roughly estimate *N*_m_ based on a qualitative inspection of a sample of interest.

Materials with (I) low, (II) medium and (III) large size polydispersity, corresponding to approximate ranges of IQR% ≤ 30, 30 < IQR% ≤ 55 and 55 < IQR% ≤ 80, respectively, are distinguished. Category I requires measuring 35 and 150 particles to reach *U*_N_ = 10% and 5%, respectively. These materials are typically colloidally stable suspensions that are monodisperse in particle size and shape (e.g. near-spherical, rod-like). In EM images, there is typically no or very few overlap of the constituent particles (only touching), which makes particle separation easily feasible by standard image analysis software. However, in the case where particle overlap is high, for instance for ERM-FD103 (see Fig. B3, Supplementary Information B), manual removal of particles detected as agglomerates or aggregates still leads to a narrow size distribution and low *N*_m_. Category II and III materials are typically provided as powders. Category II requires measuring 110 and 450 particles to reach *U*_N_ = 10% and 5%, respectively. Their constituent particles are similar in shape (e.g. near-spherical) but vary considerably in size and, moreover, they form some aggregates and agglomerates. Small agglomerates are typically well-separated by the image analysis algorithm, but within larger agglomerates or aggregates with a lot of overlap multiple particles are often detected as one. This results in *N*_m_ values considerably higher than category I materials. Category III requires measuring 260 and 1000 particles to reach *U*_N_ = 10% and 5%, respectively, and they typically consist of strongly aggregated materials that have constituent particles of various and irregular shapes. Due to the material complexity, it is very difficult for conventional algorithms to reliably separate, identify and measure the external dimensions of the constituent particles within agglomerates and aggregates. For the analysis of NM-103 and NM-212, it was therefore chosen to mostly consider isolated individual particles within the size measurement (‘Single particle’ mode algorithm) and to exclude agglomerates. The large intrinsic size variation of these materials leads to the need for relatively high values of *N*_m_. For BaSO_4_RM, the irregular watershed algorithm could be applied to separate irregular particles within agglomerates and aggregates. Nevertheless, for various cases, some overlapping particles were erroneously detected as one single particle. This introduces an additional ‘artificial’ component to the size distribution, superimposed on the intrinsic size distribution width, resulting in the highest IQR% and *N*_m_ values.

For particle shape, only two material categories could be distinguished based on the data: (I) materials with low shape polydispersity IQR% ≤ 30 and (II) materials with medium shape polydispersity 30 < IQR% ≤ 55. While the same type of power law relation holds for size and shape, the distribution of materials on the curve is different since variations in size and shape parameters are not necessarily correlated. Near-spherical TiO_2_ materials (NM-100 and TiO_2_RM), for example, have a medium size polydispersity and require measuring approximately 100 particles to reach *U*_N_ = 10%. On the other hand, they have a low shape polydispersity and determining the shape with the same precision only requires measuring 50 particles. For materials with particle shapes significantly deviating from sphericity, the position of the material on the power law curve can depend on the considered size measurand. Considering for example MICD and Fmin, NM-103 and NM-212 fall on the curve together with NM-100 and TiO_2_RM around medium polydispersity, while when considering Fmax and ECD, they fall more closely to BaSO_4_RM in the range of highly polydisperse materials. This implies that MICD and Fmin are the most robust descriptors for particle size, and thus require less particles to be measured.

### Extension to other distribution percentiles

**Fig. 5:**
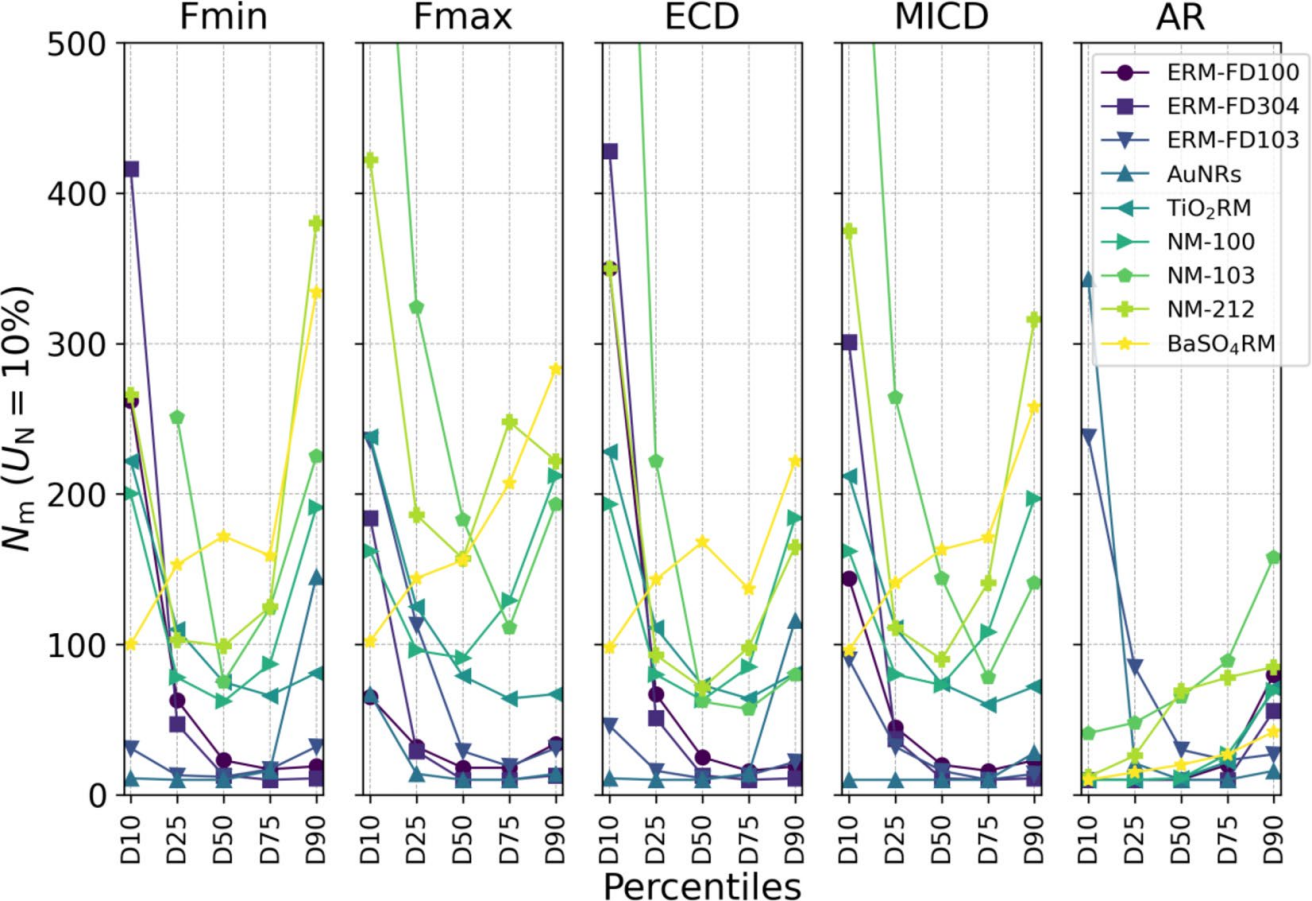
The minimum particle count to reach *U*_*N*_ *=* 10% on the measurement of the different percentiles of size and shape measurands. The straight lines between the individual values are provided only as a guide to the eye. Values for NM-103 that fall out of the plot range amount 754 (Fmax), 769 (ECD) and 1007 (MICD).

Setting *N*_m_ (*U*_N_ = 10%) as a function of the different percentiles of the size and shape PSDs results in an asymmetrical U-shape variation over the different percentiles (Fig. 5). This trend, which was found for most materials and measurands, indicates that the analysis of a greater number of particles is typically required to reach the same *U*_N_ precision of the measurement of D25 or D75 than that of D50, and even greater for D10 and D90. For other percentiles than D50, the correlation between *N*_m_ and IQR% does not necessarily hold anymore (see Supplementary Information D), explaining the crossing of material-specific curves going towards D10 and D90 in Fig. 5. This behaviour can be related to specificities of the PSDs. Especially high values of *N*_m_ are required for D10 in case of a tail on the left side (left-skewed) of the particle size distribution of the measurand, e.g. as the case for Fmin of ERM-FD304, as shown in Fig. B2 in Supplementary Information B. Relatively high values of *N*_m_ are required for D90 in case of a tail on the right side (right-skewed) of the particle size distribution of the measurand, e.g. as the case for Fmin of NM-212, as shown in Fig. B8 in Supplementary Information B. BaSO_4_RM does, however, not follow the asymmetrical U-shape trend as *N*_m_ mainly increases from D10 towards D90 for all size measurands. This can be explained by the specific shape of the size distribution, where the mode is strongly shifted towards D10, as shown in Fig. B9 in Supplementary Information B. For the same material, values of *N*_m_ (*U*_N_ = 10%) are similar between all four size measurands. For percentiles other than D50, effects related to the applied imaging conditions might have an influence on the PSD and thus on *N*_m_. It is shown, for example, for ERM-FD100 that imaging at a higher magnification leads to an improved identification of small background artefacts, which makes a more efficient estimate of D10 and D25 possible (see Supplementary Information F).

In case of the aspect ratio, the behaviour of *N*_m_ over the different percentiles relates strongly to the particle shape. For rod-like particles, like ERM-FD103 and AuNRs, a measurement of D10 with *U*_N_ = 10% requires approximately 10 times more particles than for other percentiles, due to left-skewedness of the histogram. For near-spherical particles, such as ERM-FD100, ERM-FD304, TiO_2_RM, NM-100 and BaSO_4_RM, less than 30 particles are required for D10, D25, D50 and D75 and less than 90 particles for D90. For materials with a strong shape variation, such as NM-103 and NM-212, *N*_m_ needs to be two times higher than for near-spherical particles. The values portrayed in this plot as well as the values for *U*_N_ = 5% are reported in table format in Supplementary Information G.

### *U*_*N*_ in relation to measurement uncertainty

**Fig. 6:**
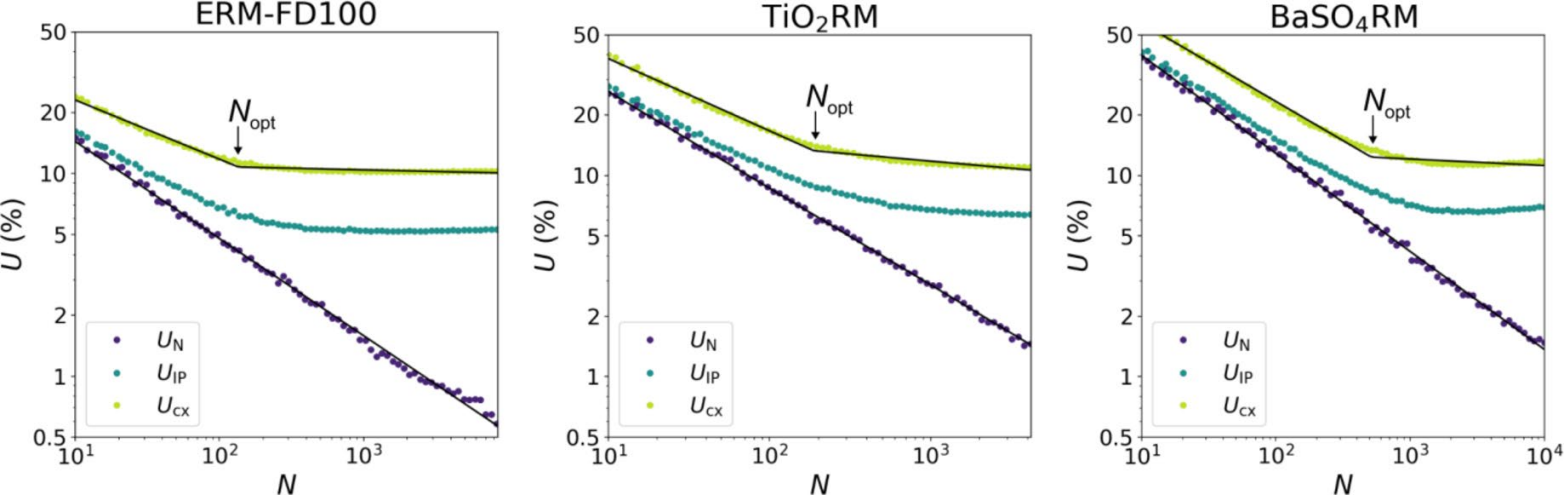
*U*_N_, *U*_IP_ and *U*_cx_ on D50 of the Fmin as a function of *N* for ERM-FD100, TiO_2_RM and BaSO_4_RM.

Figure 6 shows the behaviour of *U*_N_, *U*_IP_ and *U*_cx_ (all at *k* = 2 and for Fmin, D50) as a function of the particle count, for three selected materials: ERM-FD100, TiO_2_RM and BaSO_4_RM with relatively low, middle and high IQR%, respectively. By measuring more particles, *U*_N_ can be brought towards zero, independent of the type of material. This makes sense since the simulations resulting in *U*_N_ are produced using a single dataset, meaning that uncertainties related to sample preparation, imaging and analysis are in no way included. To include all of those, one has to look at the intermediate precision (i.e. within-day and day-to-day variation). For the lowest *N* values, *U*_N_ makes up the major part of *U*_IP_. This suggests that under these conditions, the primary source of variation between samples is the limited number of particles measured. When *N* increases, the contribution of *U*_N_ to *U*_IP_ starts to decrease. Eventually, *U*_IP_ values saturate and measuring more particles has almost no influence anymore. Variations in the measured value due to sample preparation (e.g. small variations in homogeneity, differences in particle agglomeration, …), imaging and analysis pose a limit to the uncertainty that cannot be further reduced by measuring more particles. Both *U*_N_ and *U*_IP_ give a larger contribution for high IQR% materials, demonstrated by the upward shift of their curves from ERM-FD100 towards BaSO_4_RM. The behaviour of the total uncertainty as a function of *N* is dictated by that of the intermediate precision. From a certain value of *N*, defined by *N*_opt_, the curve falls back to a smaller decreasing slope. For *N* > *N*_opt_, measuring more particles will only lead to a marginal decrease in *U*_cx_. *N*_opt_ is quantified by the piecewise linear fit, as overlaid on the data in Fig. 6. The values of *N*_opt_ for D50 as well as for the other percentiles (for Fmin), as determined in the same way, are tabulated in Table 3, together with the corresponding values of *U*_cx_. It is clear that *N*_opt_, just like *N*_m_, scales with IQR%. For materials with higher polydispersity, more particles need to be measured before the uncertainty becomes limited by other contributions. The way *N*_opt_ changes with the different percentiles depends again on the specific shape of the measurand distribution, and behaves similarly as *N*_m_ behaves with the percentiles in Fig. 5. The total expanded uncertainties that can be reached in a cost-effective way fluctuate just above 10% or sometimes towards 20% for the outer percentiles. These values take into account all sources of uncertainty, and assure precise results. The reported *N*_opt_ values are therefore systematically higher than *N*_m_ values shown in Fig. 5.

**Table 3:** *N*_opt_ and corresponding *U*_cx_ values (for Fmin) for three materials of varying IQR%.

	ERM-FD100		TiO_2_RM		BaSO_4_RM	
	*N*_opt_	U_cx_	*N*_opt_	U_cx_	*N*_opt_	U_cx_
D10	405	19%	285	18%	897	8%
D25	140	15%	219	16%	369	13%
D50	136	11%	189	13%	513	12%
D75	86	11%	235	12%	439	12%
D90	193	11%	261	12%	575	14%

### Limitations and future directives for improvement

While TEM is a powerful tool for nanomaterial characterization in a regulatory context, it has inherent limitations that must be considered. A first limitation originates from the fact that the particles have to be transferred onto a support (e.g. TEM grid) in a representative manner. The applied sampling method may introduce a bias, which is not accounted for in the measurement uncertainty estimated through repeated application of the same procedure, as done in the selected validation approach. Similarly, potential biases in the image analysis method are not considered. Additionally, the trueness of the TEM method could not be validated for the more complex materials due to the lack of suitable reference materials certified for particle dimensions.

Another key limitation is that TEM generates 2D images of 3D (nano)particles. This limitation is less pronounced for isotropically shaped particles, such as the majority of those included in our study, where a 2D projection is a satisfactory representation of their 3D size. However, for anisotropic particles, such as plate- or rod-like particles, preferential orientation on the grid can lead to biased measurements, compromising an accurate measurement of the minimum external dimension. To overcome this challenge, one should consider adapted sample preparation approaches or employ complementary EM-based techniques such as 3D imaging via tomography or thickness measurements.

Furthermore, diffraction effects can introduce contrast variations that impact the identification and segmentation of particles. For instance, Fig. B3 in Supplementary Information B illustrates the difficulty in reliably identifying rods with low diffraction contrast compared to the background, while Fig. B6 in Supplementary Information B demonstrates how diffraction contrast variations within particles can lead to incorrect segmentation. These challenges underscore the limitations of TEM for characterizing materials with strong diffraction contrast, highlighting the potential benefits of alternative techniques such as annular dark-field scanning transmission electron microscopy (ADF-STEM), which provides an incoherent imaging mechanism and enhanced contrast for materials with high atomic number^43^.

Figure 4 illustrates that the width of the particle size distribution strongly determines *N*_m_. The segmentation algorithm’s performance is another factor that can impact *N*_m _as it can artificially broaden the intrinsic particle size distribution (as discussed earlier). This effect, particularly expected for complex materials, can be reduced by further optimization of sample preparation to reduce particle agglomeration/aggregation and/or by applying more advanced segmentation algorithms, such as artificial intelligence (AI)-based methods^44–48^, to improve accuracy and further optimize *N*_m_.

The optimization of *N*_m _is an important step toward more efficient TEM measurements. However, high-throughput automated TEM analysis is still a challenge due to the need for manual sample loading and limitations of TEM sample holders. While a few TEM models support multiple sample storage^49,50^, they are typically restricted to a small number of samples due to space constraints in the TEM column. Additionally, automating imaging for sequential sample analysis remains challenging, particularly in the aspect of auto-focus. Nevertheless, progress towards automation has been made through the implementation of automated image acquisition software^51,52 ^and the development of AI-based image analysis tools for NP segmentation^44–48^.

### Impact

#### Improving international guidelines and standards

The results presented in this contribution can refine international guidelines and documentary standards that currently implement a more conservative and material- and measurand- independent approach (e.g. OECD TG 125, ISO 21363, ISO 19749). OECD TG 125 only takes into account a distinction between materials with narrow and wide size distributions and requires measurement of respectively 300 and 700 particles. ISO 21363 and ISO 19749 propose counting of at least 500 particles. These instructions are based solely on theoretical considerations, not taking into account sampling and methodological variations. In addition, the standards do not specify the obtained uncertainty in relation to particle count and measurand type. In contrast, our method allows an *ex ante* determination of the minimum particle count to reach a given level of *U*_N_ (also not taking into account sampling and methodological variations or accuracy considerations), in a material- and measurand-specific way. According to the D50 data, even for highly polydisperse materials, results with a predefined acceptable precision of 10% can be obtained by measuring 300 or less (constituent) particles, which is less than prescribed in the current guidelines. To reach a precision of 5%, less than 150 to 450 particles are sufficient for materials with low to medium size polydispersity (IQR% ≤ 55), while 1000 particles are needed for materials with large size variation (55 < IQR% ≤ 80). For other percentiles, *N*_m_ can vary strongly depending on the shape of the distribution and might be either higher or lower than the values prescribed by OECD and ISO guidelines.

The precision determined by *U*_N_ forms an important indication for evaluating fluctuations or trends in product quality. Regulatory compliance, on the other hand, requires unbiased or accurate assessments and, therefore, knowledge of the precision set by *U*_N_ alone is normally not enough. Taking into account intermediate precision and accuracy components, and looking at the behaviour of *U*_cx_ as a function of *N*, shows that these contributions pose a limit to the reachable measurement uncertainty. For representative low, medium and high IQR% materials, the total uncertainty of the median Fmin value can be improved only marginally for particle counts exceeding *N*_opt_ = 136, 189 and 513, respectively. The same is true for other percentiles, where the values of *N*_opt_ are typically higher than that of D50 but depend on the exact shape of the distribution.

Thus, in case of (i) complex materials, where image analysis might require a (partially) manual procedure due to irregular particle shapes and/or particle aggregation/agglomeration and overlap, and/or (ii) low numbers of particles per field of view, e.g. due to a low particle concentration after extraction from a matrix, a better time- and cost-efficiency can be achieved, both in terms of imaging and analysis time, than proposed by current guidelines. Updating these guidelines can serve as a basis to update existing EFSA and ECHA guidance^18–20^ for assessing the presence of a fraction of nanoparticles in food and feed products and determining whether a chemical substance is a nanoform, respectively.

#### Implementation of legislation

The outcome of this work can contribute to the implementation of legislation requiring precise measurement of characteristic values of particle size and shape distributions. The method does not take into account possible significant biases due to sampling or image analysis. Measurement of the median (D50) of the number-based distribution of the particle’s minimum external dimension, using Fmin, MICD or ECD as proxy-measurands, allows to implement the EU legislation that specifically defines and addresses nanomaterials. In this perspective, our results underpin selecting the 50% threshold (D50) that is used in the EC’s Recommendation on the definition of nanomaterial (2022/C 229/01)^15^, because for most examined materials less particles need to be measured for precisely estimating D50 than for measuring other percentiles with the same precision. For materials with irregular particle shapes and heterogeneous shape variation, the efficiency of the analysis can benefit from the choice of the size measurand (e.g. MICD) to quantify the minimum external dimension, as required by the EC nanomaterial definition.

Our work can also impact EM characterizations carried out in the context of risk assessment. For the use of NM in food products, the EFSA guidance TG-RA^19 ^prescribes nanomaterial-specific risk assessments based on measurement of the 10%-fraction of smallest particles within the sample. Our results underline the need to measure more particles to achieve the same uncertainty on D10 (or other percentiles) as on D50. For high aspect ratio nanoparticles, precise estimation of the aspect ratio and its distribution is relevant because particle shape is known to influence the particle toxicity, especially in the context of inhalation^20,53^.

## Conclusions

Based on particle data obtained from validated TEM methods, a strategy is proposed to determine the precision specifically related to particle count *U*_N_ to measure characteristic values of the number-based distributions of particle size and shape measurands. This empirical method was evaluated for a variety of reference and test materials with variable size and shape distributions, degrees of polydispersity and agglomeration properties. The established log-log relationship between the minimum particle count *N*_m_ and the associated precision enables both *ex ante* and *ex post* estimations of *N*_m_ based on the chosen precision level, incorporating material-specific characteristics and image analysis considerations. The log-log nature of the relationship implies that a significant increase in the number of particles is required to achieve a reduction in precision. To determine the median of Fmin, Fmax, ECD, MICD and AR, the measurand’s distribution width was shown to be the most relevant parameter determining *N*_m_. As a generalized rule, materials with respective IQR% ranges of IQR% ≤ 30, 30 ≤ IQR% < 55 and 55 ≤ IQR% < 80 require a minimum particle count of 150, 450 and 1000 to reach *U*_N_ = 5 % for the measurement of the median.

While *U*_N_ provides an important measure of precision for evaluating trends and product quality, it is insufficient to assess measurements used for regulatory purposes, which requires unbiased and accurate assessments. Taking into account components related to intermediate precision, calibration uncertainty and trueness uncertainty to determine the total uncertainty as a function of *N*, it was demonstrated that these factors limit the attainable uncertainty. This defines an optimal value of particle count above which measuring more particles becomes ineffective.

Our findings can refine established guidelines, such as OECD TG 125, ISO 21363 and ISO 19,49, which are based on log-normal simulations and give conservative and generalized estimates for the required particle count. Our research is relevant for the implementation of and compliance with legislation specifically addressing nanomaterials and guidance documents requiring characterization of nanomaterials, e.g. EFSA guidance for assessing the presence of fractions of nanoparticles in food and feed and determining whether a chemical substance is a nanoform under REACH. Risk assessment of nanotechnology can also benefit from these results, allowing more cost- and time-efficient analyses especially in complex or manual measurements. Ultimately, our findings provide a comprehensive and adaptable framework for optimizing particle measurements in diverse contexts, enhancing accuracy and resource utilization.

## Electronic supplementary material

Below is the link to the electronic supplementary material.

Supplementary Material 1

## Data Availability

The python code that was applied to calculate the minimum particle count as a function of precision can be found on Github: https://github.com/chawou/NumPar. Users can use the NumPar code to perform the calculations for their own materials, given a particle number-based measurement dataset. The material datasets used and analyzed during the current study are available from the corresponding author on reasonable request.

## Abbreviations

AI: Artificial intelligence
ADF-STEM: Annular dark field scanning transmission electron microscopy
AR: Aspect ratio
ECHA: European Chemicals Agency
ECD: Area-equivalent circular diameter
EFSA: European Food Safety Authority
EM: Electron microscopy
EC: European Commission
EU: European Union
Fmin: Minimum Feret diameter
Fmax: Maximum Feret diameter
IQR: Interquartile range
IQR%: Interquartile range divided by the median of the distribution and multiplied by 100
ISO: International Organization for Standardization
JRC: Joint Research Centre
MICD: Maximum inscribed circular diameter
*N*_m_: Minimum particle count
*N*_opt_: Particle count for which the decrease in *U*_cx_ becomes limited by various uncertainty contributions
OECD: Organisation for Economic Co-operation and Development
PSD: Particle size/shape distribution
REACH: (EU Regulation on) Registration, Evaluation, Authorisation and Restriction of Chemicals
SEM: Scanning electron microscopy
TEM: Transmission electron microscopy

## References

[1] Baig, N., Kammakakam, I. & Falath, W. Nanomaterials: a review of synthesis methods, properties, recent progress, and challenges. *Mater. Adv.* **2**, 1821–1871 (2021).

[2] Gupta, V. et al. Nanotechnology in cosmetics and Cosmeceuticals—A review of latest advancements. *Gels* **8**, 173 (2022). 35323286 10.3390/gels8030173 PMC8951203

[3] Hofmann, T. et al. Technology readiness and overcoming barriers to sustainably implement nanotechnology-enabled plant agriculture. *Nat. Food*. **1**, 416–425 (2020).

[4] Ameta, S. K., Rai, A. K., Hiran, D., Ameta, R. & Ameta, S. C. Use of nanomaterials in food science. in Biogenic Nano-Particles and their Use in Agro-ecosystems (eds Ghorbanpour, M., Bhargava, P., Varma, A. & Choudhary, D. K.) 457–488 (Springer, Singapore, doi:10.1007/978-981-15-2985-6_24. (2020).

[5] Mast, J. et al. Application of silver-based biocides in face masks intended for general use requires regulatory control. *Sci. Total Environ.* **870**, 161889 (2023). 36731552 10.1016/j.scitotenv.2023.161889 PMC9886386

[6] Ruggeri, M. et al. Nanotechnology-Based medical devices for the treatment of chronic skin lesions: from research to the clinic. *Pharmaceutics* **12**, 815 (2020). 32867241 10.3390/pharmaceutics12090815 PMC7559814

[7] Regulation No. 1223/2009 of the European Parliament and of the Council of 30 November 2009 on cosmetic products. *Official J. Eur. Union* **L342/59**, (2009).

[8] Regulation, E. C. 1333/2008 of the European Parliament and of the Council of 16 December 2008 on food additives. *Official J. Eur. Union* **L 354/16**, (2008).

[9] Regulation 528/2012 of the European Parliament and of the Council of 22. May 2012 concerning the making available on the market and use of biocidal products. *Official J. Eur. Union* **L167/1**, (2012).

[10] *Proposal for a Regulation of the European Parliament and of the Council on Medical Devices, and Amending Directive 2001/83/EC, Regulation No 178/2002 and Regulation No 1223/2009, COM(2012) 542 Final*. (2012).

[11] Commission Regulation (EU). 10/2011 of 14 January 2011 on plastic materials and articles intended to come into contact with food. *Official J. Eur. Union* **L12/1**, (2011).

[12] Regulation 2015/2283 of the European Parliament and of the Council of 25 November. on Novel Foods, Amending Regulation (EU) No 1169/2011 of the European Parliament and of the Council and repealing Regulation (EC) No 258/97 of the European Parliament and of the Council and Commission Regulation (EC) No 1852/2001. *Official Journal of the European Union* L327/1, (2015).

[13] of 3 December 2018 amending Regulation (EC) No 1907/2006 of the European Parliament and of the Council on the Registration, Evaluation, Authorisation and Restriction of Chemicals (REACH) as regards Annexes I, Commission Regulation (EU) & III,VI, V. I. I. VIII, IX, X, XI, and XII to address nanoforms of substances (Text with EEA relevance.). *Official Journal of the European Union* L 308, 1–20 (2018). (2018)/1881.

[14] Commission Recommendation of 18 October 2011 on the definition of nanomaterial. *Official J. Eur. Union* **L 275**, 38–40 .

[15] Commission Recommendation of 10 June 2022 on the definition of nanomaterial 2022/C 229/01. *Official J. Eur. Union* **C 229**, 1–5 (2022).

[16] Rasmussen, K., Riego Sintes, J. & Rauscher, H. How nanoparticles are counted in global regulatory nanomaterial definitions. *Nat. Nanotechnol*. **19**, 132–138 (2024). 38308175 10.1038/s41565-023-01578-x

[17] Bresch, H., Hodoroaba, V. D., Schmidt, A., Rasmussen, K. & Rauscher, H. Counting small particles in Electron microscopy Images—Proposal for rules and their application in practice. *Nanomaterials* **12**, 2238 (2022). 35808073 10.3390/nano12132238 PMC9268650

[18] Scientific Committee, E. F. S. A. et al. Guidance on technical requirements for regulated food and feed product applications to Establish the presence of small particles including nanoparticles. *EFSA J.* **19**, e06769 (2021). 34377191 10.2903/j.efsa.2021.6769 PMC8331058

[19] EFSA Scientific Committee. et al. Guidance on risk assessment of nanomaterials to be applied in the food and feed chain: human and animal health. *EFSA J.* **19**, (2021). 10.2903/j.efsa.2021.6768 PMC8331059 34377190

[20] ECHA. Appendix for nanoforms to the guidance on registration and the guidance on substance identification: version 2.0 January 2022. https://doi.org/10.2823/40. (2022).

[21] Rauscher, H. et al. *Identification of nanomaterials through measurements.* Luxembourg: Publications Office of the European Union; (Report EUR 29942), ISBN 978-92-76-10372-1, 2019 10.2760/7644, JRC118158. 10.2760/053982.

[22] Rauscher, H. *et al. An Overview of Concepts and Terms Used in the European Commission’s Definition of Nanomaterial. Luxembourg: Publications Office of the European Union; 2019 (Report EUR 29647), ISBN 978-92-79-99660-3, Doi:10.2760/459136, JRC113469. (2019)*.

[23] Rauscher, H., Kestens, V., Rasmussen, K., Linsinger, T. & Stefaniak, E. *Guidance on the Implementation of the Commission Recommendation 2022/C 229/01 on the Definition of Nanomaterial, EUR 31452 EN, Publications Office of the European Union, Luxembourg*, ISBN 978-92-68-01243-7, Doi:10.2760/237496, JRC132102. (2023). 10.2760/143118

[24] Braun, A. et al. Validation of dynamic light scattering and centrifugal liquid sedimentation methods for nanoparticle characterisation. *Adv. Powder Technol.* **22**, 766–770 (2011).

[25] OECD. Test Guideline No. 125: Nanomaterial Particle Size and Size Distribution of Nanomaterials. (2023).

[26] ISO. Nanotechnologies — Measurements of Particle Size and Shape Distributions by Transmission Electron Microscopy. ISO 21363:2020. (2020).

[27] ISO. Nanotechnologies - Measurements of Particle Size and Shape Distributions by Scanning Electron Microscopy. ISO 19749:2021. (2021).

[28] Masuda, H. & Gotoh, K. Study on the sample size required for the Estimation of mean particle diameter. *Adv. Powder Technol.* **10**, 159–173 (1999).

[29] ISO. Particle Size Analysis — Image Analysis Methods — Part 1: Static Image Analysis Methods. ISO 13322-1:2014. (2014).

[30] Matsuyama, T. Estimation of uncertainty of percentile values in particle size distribution analysis as a function of number of particles. *Adv. Powder Technol.* **30**, 2616–2619 (2019).

[31] Dudkiewicz, A. et al. Uncertainties of size measurements in electron microscopy characterization of nanomaterials in foods. *Food Chem.* **176**, 472–479 (2015). 25624258 10.1016/j.foodchem.2014.12.071

[32] Verleysen, E. et al. Evaluation of a TEM based approach for size measurement of particulate (Nano) materials. *Materials* **12**, 2274 (2019). 31311143 10.3390/ma12142274 PMC6679035

[33] De Temmerman, P. J. et al. Measurement uncertainties of size, shape, and surface measurements using transmission electron microscopy of near-monodisperse, near-spherical nanoparticles. *J. Nanopart. Res.* **16**, 1–22 (2013).

[34] Braun, A. et al. *Certified Reference Material ERM®- FD100: Certification of Equivalent Spherical Diameters of Silica Nanoparticles in Water* (Publications Office of the European Union, 2011).

[35] Franks, K. et al. *Certified Reference Material ERM®-FD304: Certification of the Equivalent Spherical Diameters of Silica Nanoparticles in Aqueous Solution* (Publications Office of the European Union, 2012).

[36] Kestens, V., Gerganova, T., Roebben, G. & Held, A. A new certified reference material for size and shape analysis of nanorods using electron microscopy. *Anal. Bioanal Chem.* **413**, 141–157 (2021). 33048174 10.1007/s00216-020-02984-z PMC7801322

[37] Rasmussen, K. et al. Titanium Dioxide, NM-100, NM-101, NM-102, NM-103, NM-104, NM-105: Characterisation and Physico-Chemical Properties. *EUR 26637* (2014). 10.2788/79554

[38] Singh, C. et al. Cerium dioxide NM-211, NM-212, NM-213, characterisation and test item Preparation. *JRC Repository: NM-series Representative Manufactured Nanomaterials* (2014).

[39] NANoREG. Regulatory testing of nanomaterials, A common European approach to the regulatory testing of Nanomaterials. EC Large-scale integrating project FP7 2013–2017: NMP.2012.1.3-3. (2013).

[40] NanoDefine Development of an integrated approach based on validated and standardized methods to support the implementation of the EC recommendation for a definition of nanomaterial. *FP7-NMP-2013-LARGE-7: NMP.2013.1.4-3* (2013).

[41] Wagner, T. ij-particlesizer: particlesizer 1.0.9. *Zenodo*10.5281/zenodo.56457 (2016).

[42] ISO. *Representation of Results of Particle Size Analysis - Part 6: Descriptive and Quantitative Representation of Particle Shape and Morphology*. *ISO 9276-6*: (2008). (2008).

[43] Ponce, A., Mejía-Rosales, S. & José-Yacamán, M. Scanning transmission Electron microscopy methods for the analysis of nanoparticles. in Nanoparticles in Biology and Medicine: Methods and Protocols (ed Soloviev, M.) 453–471 (Humana, Totowa, NJ, doi:10.1007/978-1-61779-953-2_37. (2012). 10.1007/978-1-61779-953-2_37 22791456

[44] Monteiro, G. A. A., Monteiro, B. A. A., dos Santos, J. A. & Wittemann, A. Pre-trained artificial intelligence-aided analysis of nanoparticles using the segment anything model. *Sci. Rep.* **15**, 2341 (2025). 39825089 10.1038/s41598-025-86327-x PMC11748653

[45] Larsen, R., Villadsen, T. L., Mathiesen, J. K., Jensen, K. M. O. & Boejesen, E. D. NP-SAM: Implementing the Segment Anything Model for Easy Nanoparticle Segmentation in Electron Microscopy Images. Preprint at 10.26434/chemrxiv-2023-k73qz-v2

[46] Lin, B. et al. A deep learned nanowire segmentation model using synthetic data augmentation. *Npj Comput. Mater.* **8**, 1–12 (2022).

[47] Genc, A., Marlowe, J., Finzel, J. & Christopher, P. AI-Enhanced Nanoparticle Analysis: Integrating Single-Shot Object Detection and Vision Transformer for Rapid and Accurate Characterization. *Microscopy and Microanalysis* 30, ozae044.196 (2024).

[48] Gumbiowski, N., Loza, K., Heggen, M. & Epple, M. Automated analysis of transmission electron micrographs of metallic nanoparticles by machine learning. *Nanoscale Adv.* **5**, 2318–2326 . 10.1039/d2na00781a PMC10089082 37056630

[49] Multiple Specimen Holders for TEM | Gatan, Inc. https://www.gatan.com/products/tem-specimen-holders/multiple-specimen-holders

[50] Hitachi 3-Position Multi-Sample Holder. *Electron Microscopy Sciences* https://www.emsdiasum.com/multi-position-tilt-holders-4

[51] Schorb, M., Haberbosch, I., Hagen, W. J., Schwab, Y. & Mastronarde, D. N. Software tools for automated transmission Electron microscopy. *Nat. Methods*. **16**, 471–477 (2019). 31086343 10.1038/s41592-019-0396-9 PMC7000238

[52] Uusimaeki, T., Wagner, T., Lipinski, H. G. & Kaegi, R. AutoEM: a software for automated acquisition and analysis of nanoparticles. *J. Nanopart. Res.* **21**, 122 (2019).

[53] Jaurand, M. C. F., Renier, A., Daubriac, J. & Mesothelioma Do asbestos and carbon nanotubes pose the same health risk? *Part. Fibre Toxicol.* **6**, 16 (2009). 19523217 10.1186/1743-8977-6-16 PMC2706793

