# Supplementary material for: Assessing particle count in electron microscopy measurements of nanomaterials to support regulatory guidance
Source: Sci Rep. 2025 Apr 7;15:11803. doi: 10.1038/s41598-025-92266-4 (PMC11973222; doi:10.1038/s41598-025-92266-4)
Supplement: Supplementary file 1 — Supplementary Material 1 [file 41598_2025_92266_MOESM1_ESM.pdf]

Supplementary Information

Assessing particle count in electron microscopy measurements of nanomaterials to support regulatory guidance

Charlotte Wouters*[1], Vikram Kestens[2], Eveline Verleysen[1], Jan Mast[1]

[1]Trace Elements and Nanomaterials, Sciensano, Groeselenbergstraat 99, 1180 Uccle, Belgium
[2]European Commission, Joint Research Centre (JRC), Retieseweg 111, 2440 Geel, Belgium

A. TEM equipment and imaging conditions

Table A1 TEM microscope specifications

**Microscope**	120 kV Tecnai G2 Spirit with BioTwin lens configuration
**Detector**	Bottom-mounted 4 x 4 k Eagle CCD camera
**Acquisition software**	TEM imaging and analysis (TIA) software

All produced by Thermo Fisher Scientific, Eindhoven, The Netherlands

Table A2 TEM imaging conditions

	**ERM-FD100**	**ERM-FD304**	**ERM-FD103**	**AuNRs**	**TiO_2RM**
**Magnification for quantitative analysis**	18500x	18500x	30000x	18500x	4800x
**Pixel size (nm)**	0.598	0.598	0.373	0.585	2.24
**Number of pixels**	4096 x 4096	4096 x 4096	4096 x 4096	4096 x 4096	4096 x 4096
**Field of view (nm)**	2450 x 2450	2450 x 2450	1530 x 1530	2400 x 2400	9180 x 9180
**Lower limit of detection (LLOD) (nm)**	0.598	0.598	0.373	0.585	2.24
**Upper limit of detection (ULOD) (nm)**	2450	2450	1530	2400	9180
**Lower limit of quantification (LLOQ) (nm)**	5.98	5.98	3.73	5.85	22.4
**Upper limit of quantification (ULOQ) (nm)**	245	245	153	240	918

	**NM-100**	**NM-103**	**NM-212**	**$BaSO_4$RM**
**Magnification for quantitative analysis**	9300x	30000x	30000x	18500x
**Pixel size (nm)**	1.17	0.370	0.370	0.592
**Number of pixels**	4096 x 4096	4096 x 4096	4096 x 4096	4096 x 4096
**Field of view (nm)**	4780 x 4780	1520 x 1520	1520 x 1520	2420 x 2420
**Lower limit of detection (LLOD) (nm)**	1.17	0.370	0.370	0.592
**Upper limit of detection (ULOD) (nm)**	4780	1520	1520	2420
**Lower limit of quantification (LLOQ) (nm)**	11.7	3.70	3.70	5.92
**Upper limit of quantification (ULOQ) (nm)**	478	152	152	242

B. Representative TEM images and Fmin size distributions with percentile lines

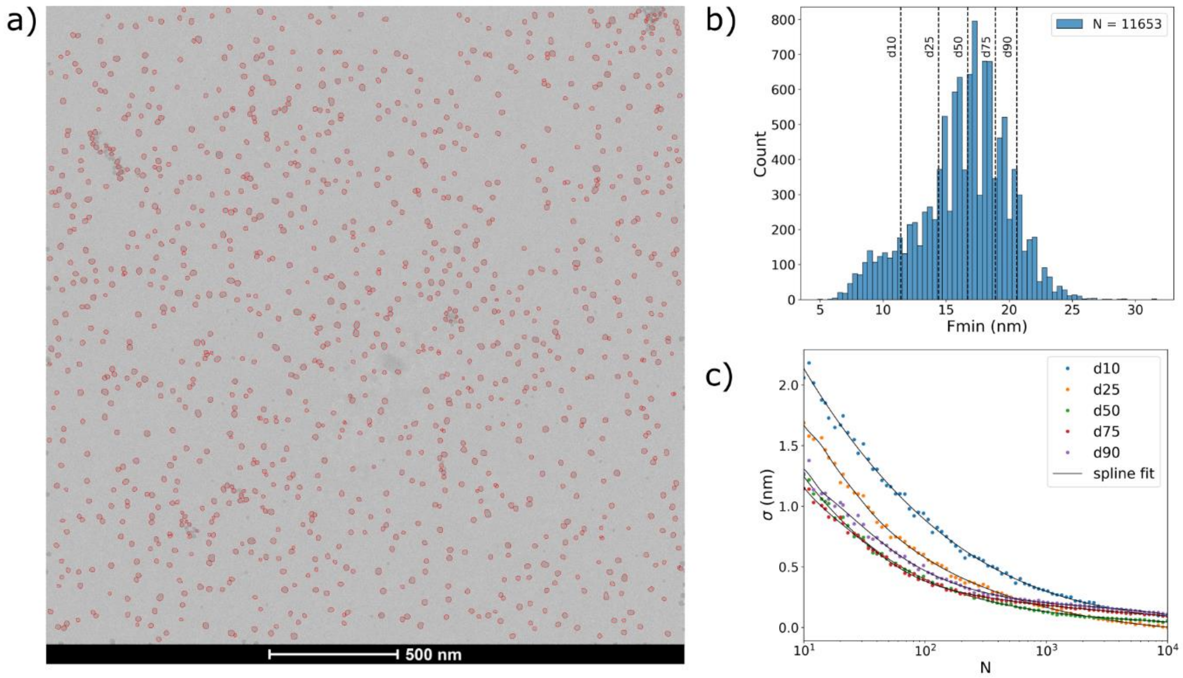

Figure B1: Representative TEM bright field image of ERM-FD100 with annotations of particles identified by ParticleSizer (a). The Fmin number-based distribution based on a series of 10 images with vertical lines indicating the percentiles (b). Variation of the standard deviation of the percentiles of the Fmin distribution of ERM-FD100 as a function of particle number N obtained by sampling 500 times at each value of N (c).

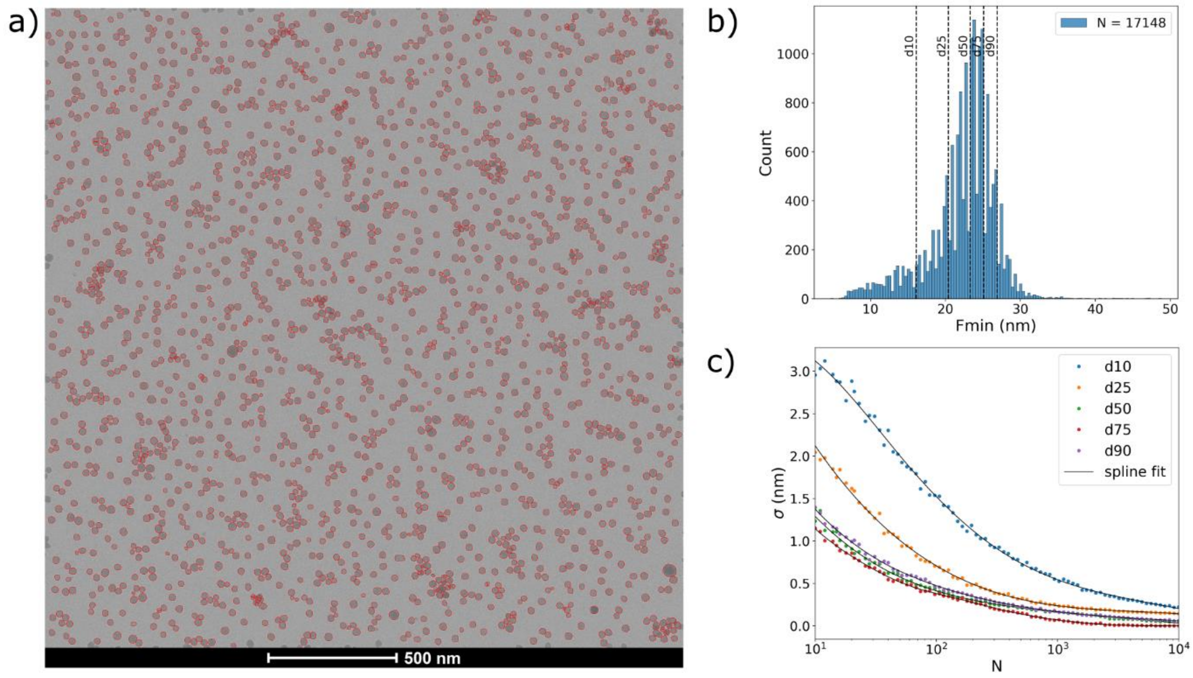

Figure B2: Representative TEM bright field image of ERM-FD304 with annotations of particles identified by ParticleSizer (a). The Fmin number-based distribution based on a series of 10 images with vertical lines indicating the percentiles (b). Variation of the standard deviation of the percentiles of the Fmin distribution of ERM-FD304 as a function of particle number N obtained by sampling 500 times at each value of N (c).

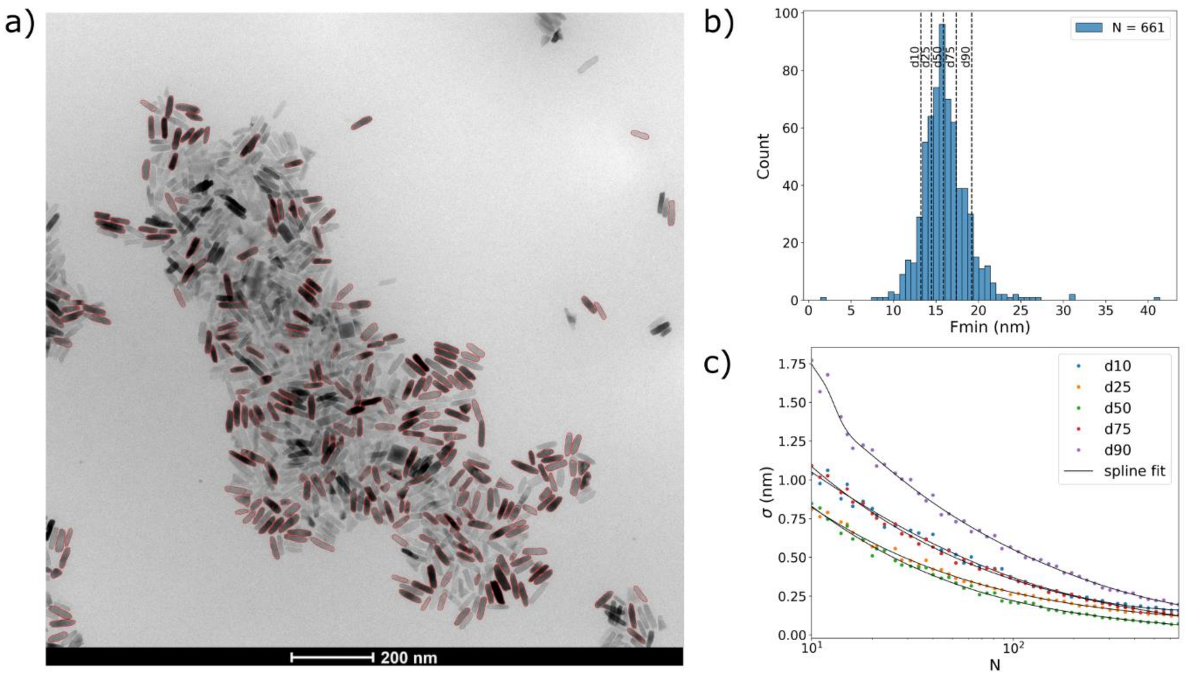

Figure B3: Representative TEM bright field image of ERM-FD103 with annotations of particles identified by ParticleSizer (a). The Fmin number-based distribution based on a series of 10 images with vertical lines indicating the percentiles (b). Variation of the standard deviation of the percentiles of the Fmin distribution of ERM-FD103 as a function of particle number N obtained by sampling 500 times at each value of N (c).

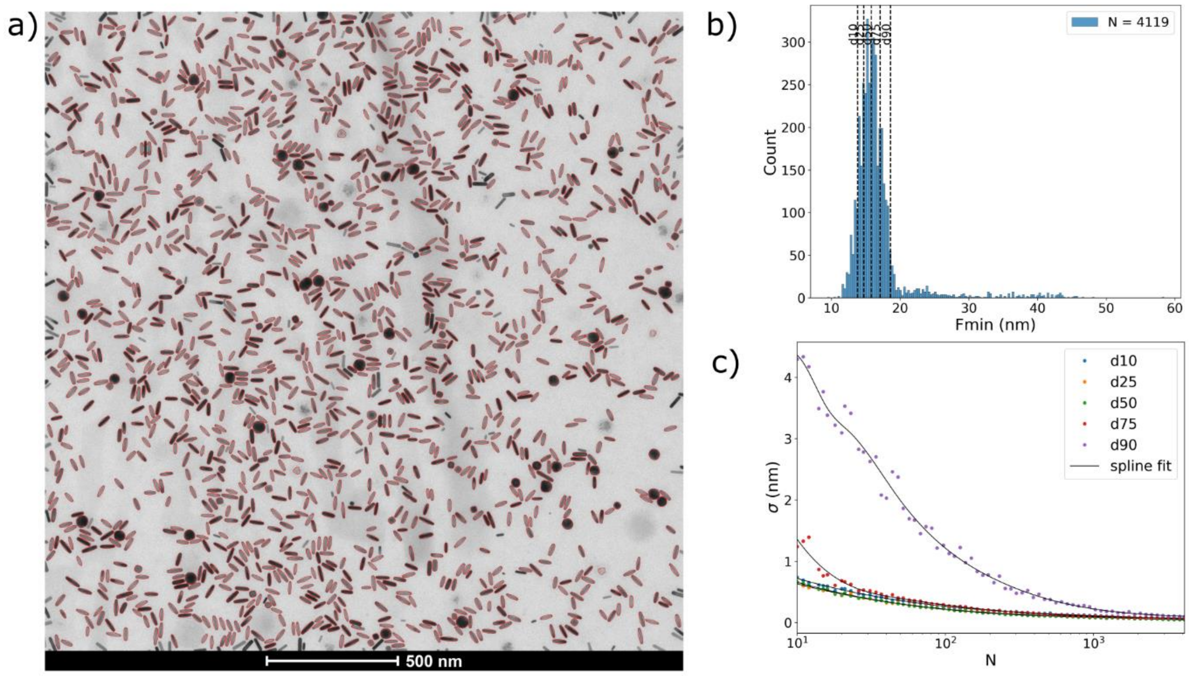

Figure B4: Representative TEM bright field image of Au nanorods with annotations of particles identified by ParticleSizer (a). The Fmin number-based distribution based on a series of 10 images with vertical lines indicating the percentiles (b). Variation of the standard deviation of the percentiles of the Fmin distribution of Au rods as a function of particle number N obtained by sampling 500 times at each value of N (c).

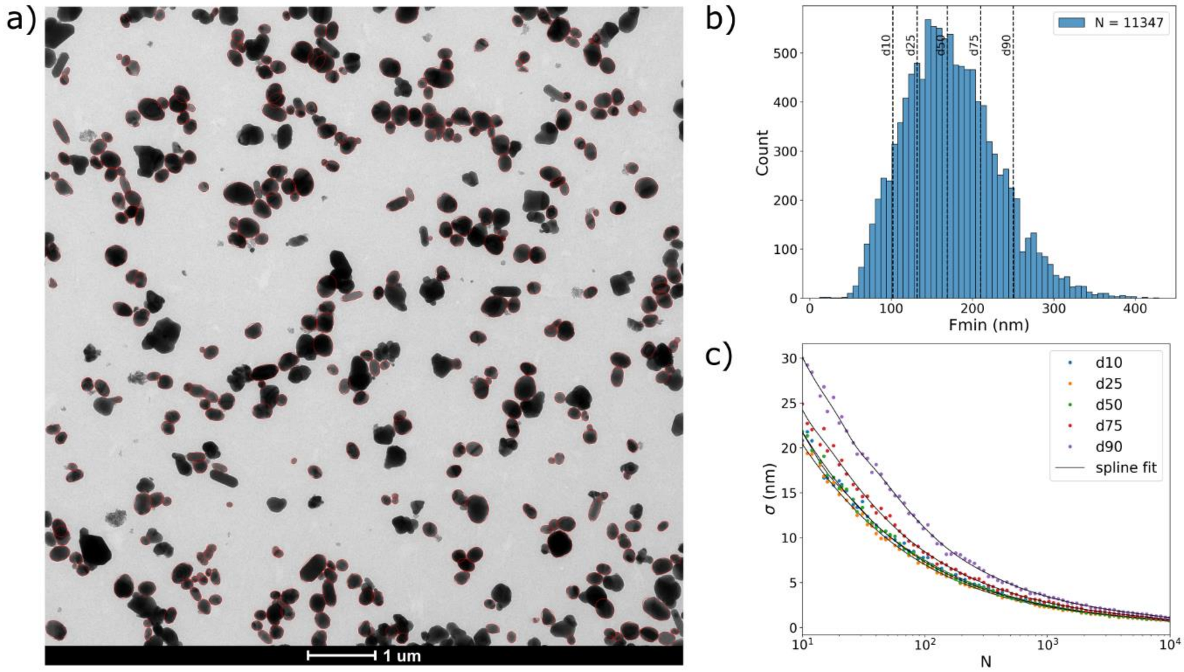

Figure B5: Representative TEM bright field image of TiO_2RM with annotations of particles identified by ParticleSizer (a). The Fmin number-based distribution based on a series of 50 images with vertical lines indicating the percentiles (b). Variation of the standard deviation of the percentiles of the Fmin distribution of TiO_2RM as a function of particle number N obtained by sampling 500 times at each value of N (c).

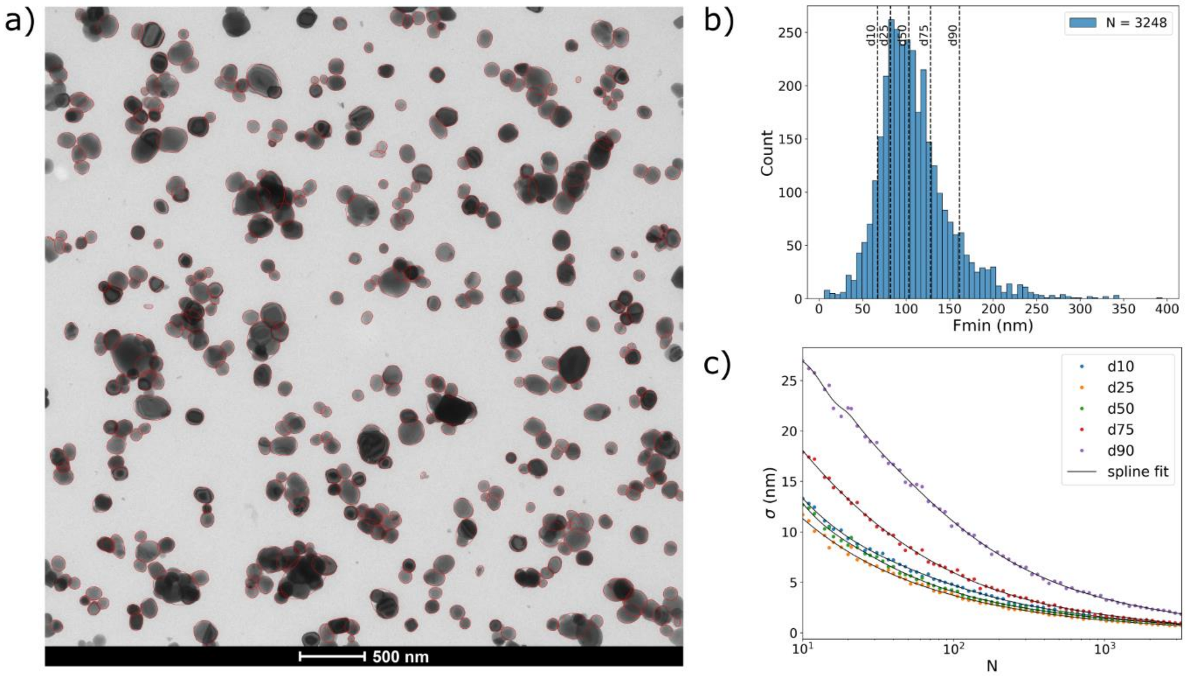

Figure B6: Representative TEM bright field image of NM100 with annotations of particles identified by ParticleSizer (a). The Fmin number-based distribution based on a series of 10 images with vertical lines indicating the percentiles (b). Variation of the standard deviation of the percentiles of the Fmin distribution of NM100 as a function of particle number N obtained by sampling 500 times at each value of N (c).

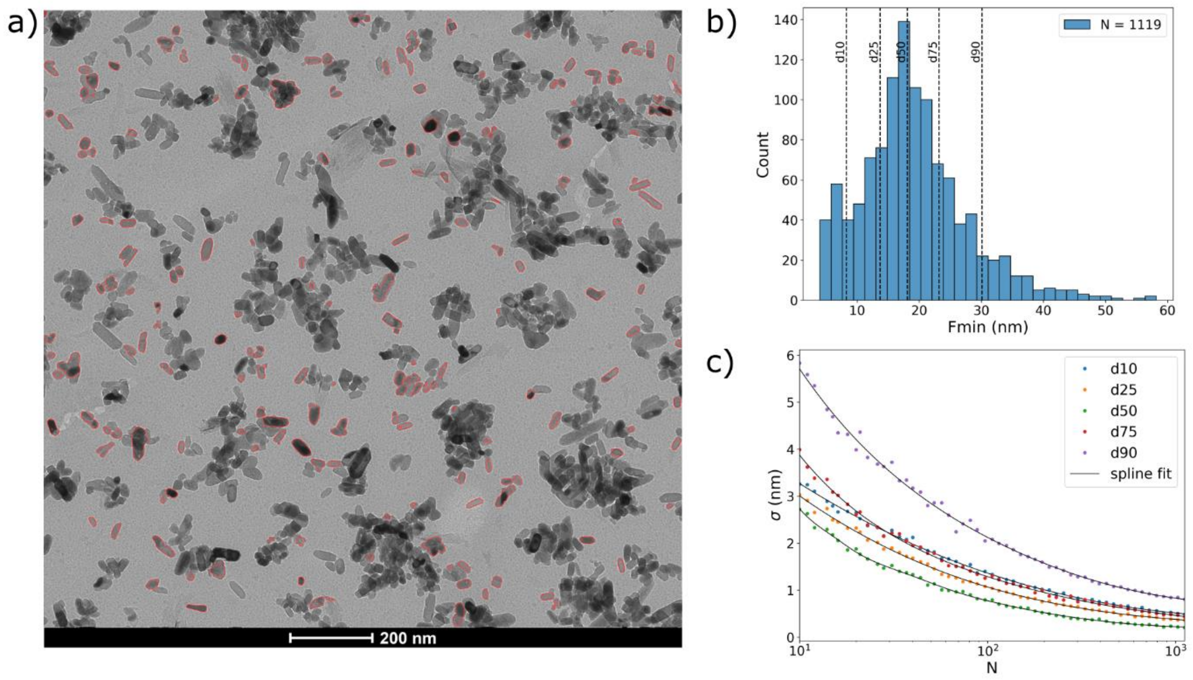

Figure B7: Representative TEM bright field image of NM103 with annotations of particles identified by ParticleSizer (a). The Fmin number-based distribution based on a series of 10 images with vertical lines indicating the percentiles (b). Variation of the standard deviation of the percentiles of the Fmin distribution of NM103 as a function of particle number N obtained by sampling 500 times at each value of N (c).

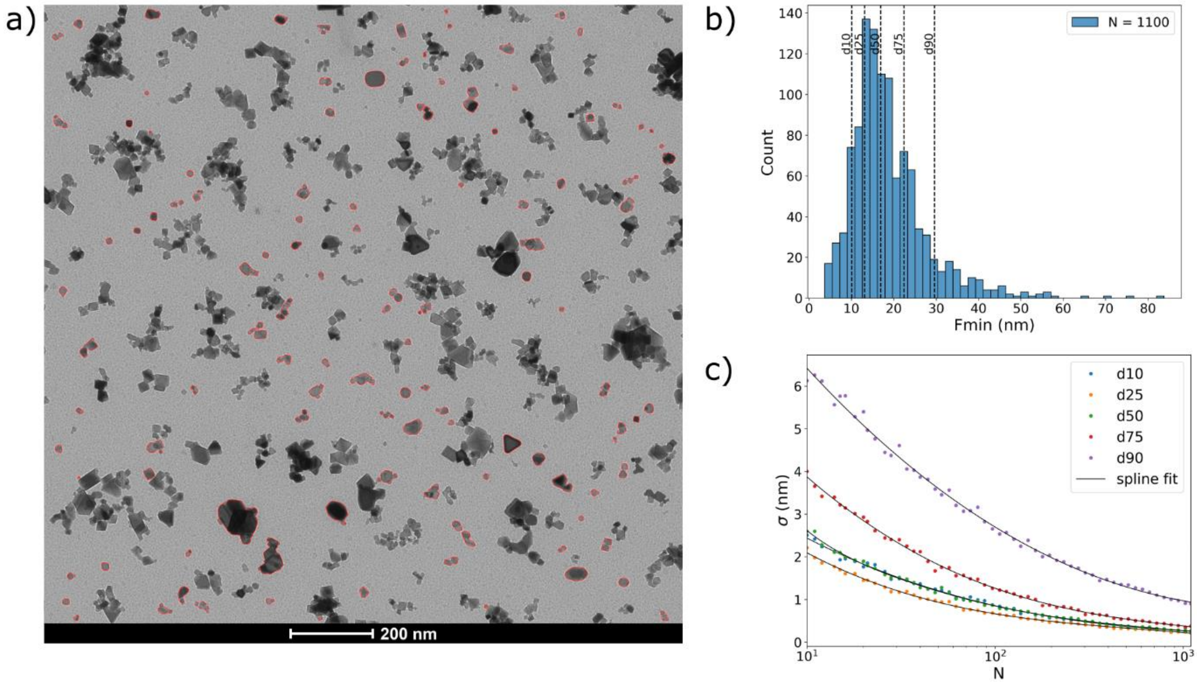

Figure B8: Representative TEM bright field image of NM212 with annotations of particles identified by ParticleSizer (a). The Fmin number-based distribution based on a series of 10 images with vertical lines indicating the percentiles (b). Variation of the standard deviation of the percentiles of the Fmin distribution of NM212 as a function of particle number N obtained by sampling 500 times at each value of N (c).

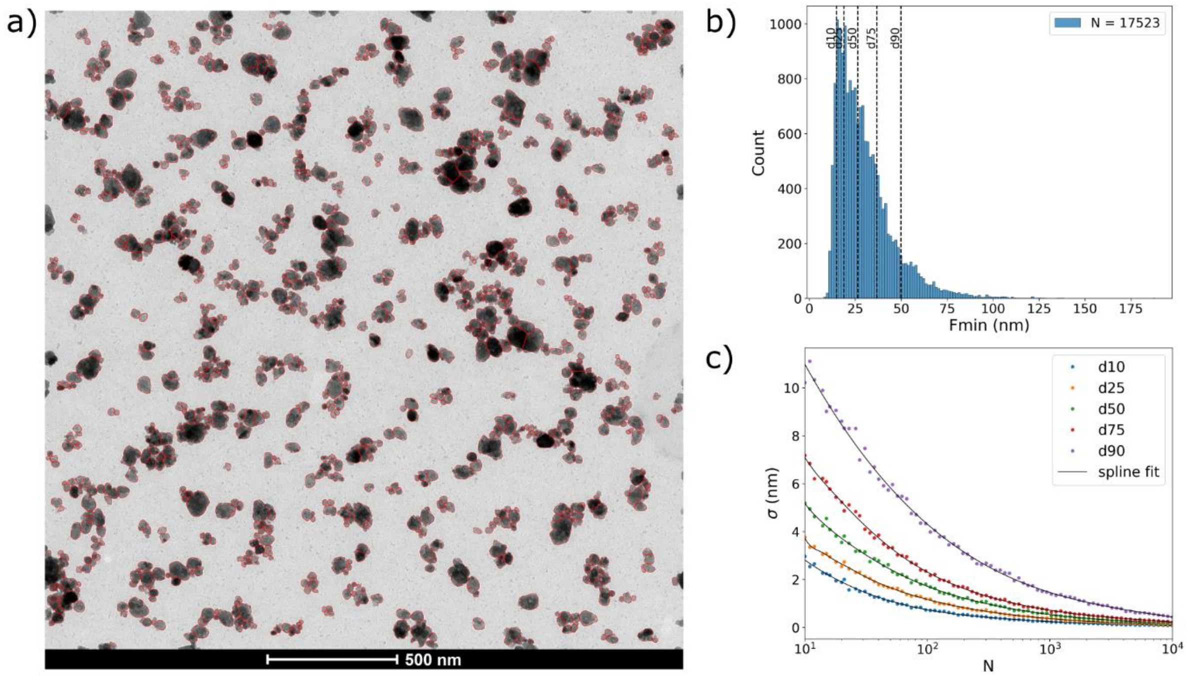

Figure B9: Representative TEM bright field image of $BaSO_4$RM with annotations of particles identified by ParticleSizer (a). b) The Fmin number-based distribution based on a series of 15 images with vertical lines indicating the percentiles (b). Variation of the standard deviation of the percentiles of the Fmin distribution of $BaSO_4$RM as a function of particle number N obtained by sampling 500 times at each value of N (c).

C. Effect of number of sampling datasets

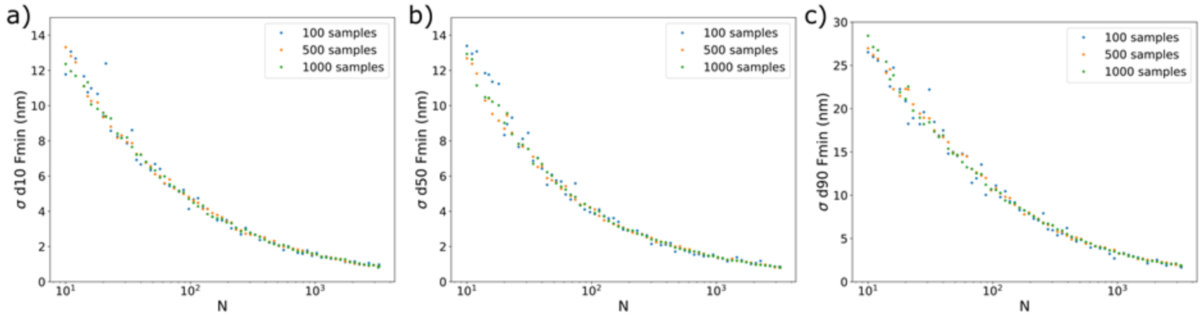

Figure C1: Variation of the standard deviation of the D10 (a), D50 (b) and D90 (c) percentiles of the Fmin distribution of NM-100 as a function of particle number N obtained by sampling 100, 500 or 1000 times at each value of N.

Figure C1 shows the influence of the number of sampling datasets taken at each value of *N* on the outcome of the performed analysis, illustrated for the case of NM-100. The standard deviation of the D10 (a), D50 (b) and D90 (c) percentiles of the Fmin distribution is compared for algorithms based on sampling 100, 500 or 1000 times. For all percentiles, the curves represented by the data points fall on top of each other, indicating consistent results. When 100 samples are considered at each *N*, there is considerably more variation/scatter in the obtained standard deviation compared to higher amount of samples. Increasing the amount of samples from 500 to 1000 samples, does not yield a significant improvement in the amount of scatter of σ. Therefore, 500 samples are considered sufficient to produce reliable data and to allow a straightforward process of finding a smooth curve through the data points.

D. N_m as a function of U_N

Fmin

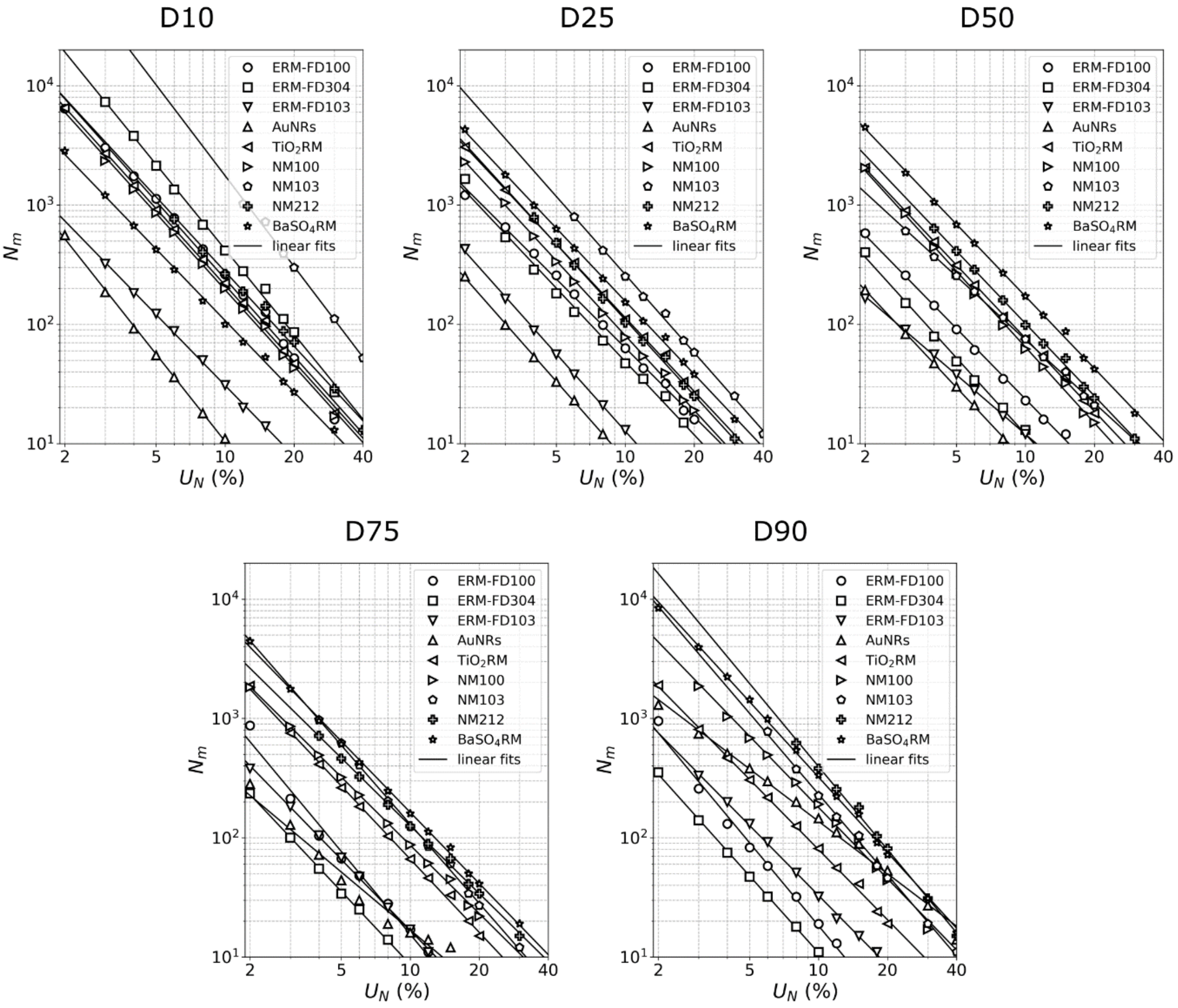

Figure D1: The minimum number of particles as a function of the desired U_N level for the measurement of the percentiles of the Fmin distribution, with linear log-log fits to the data.

Fmax

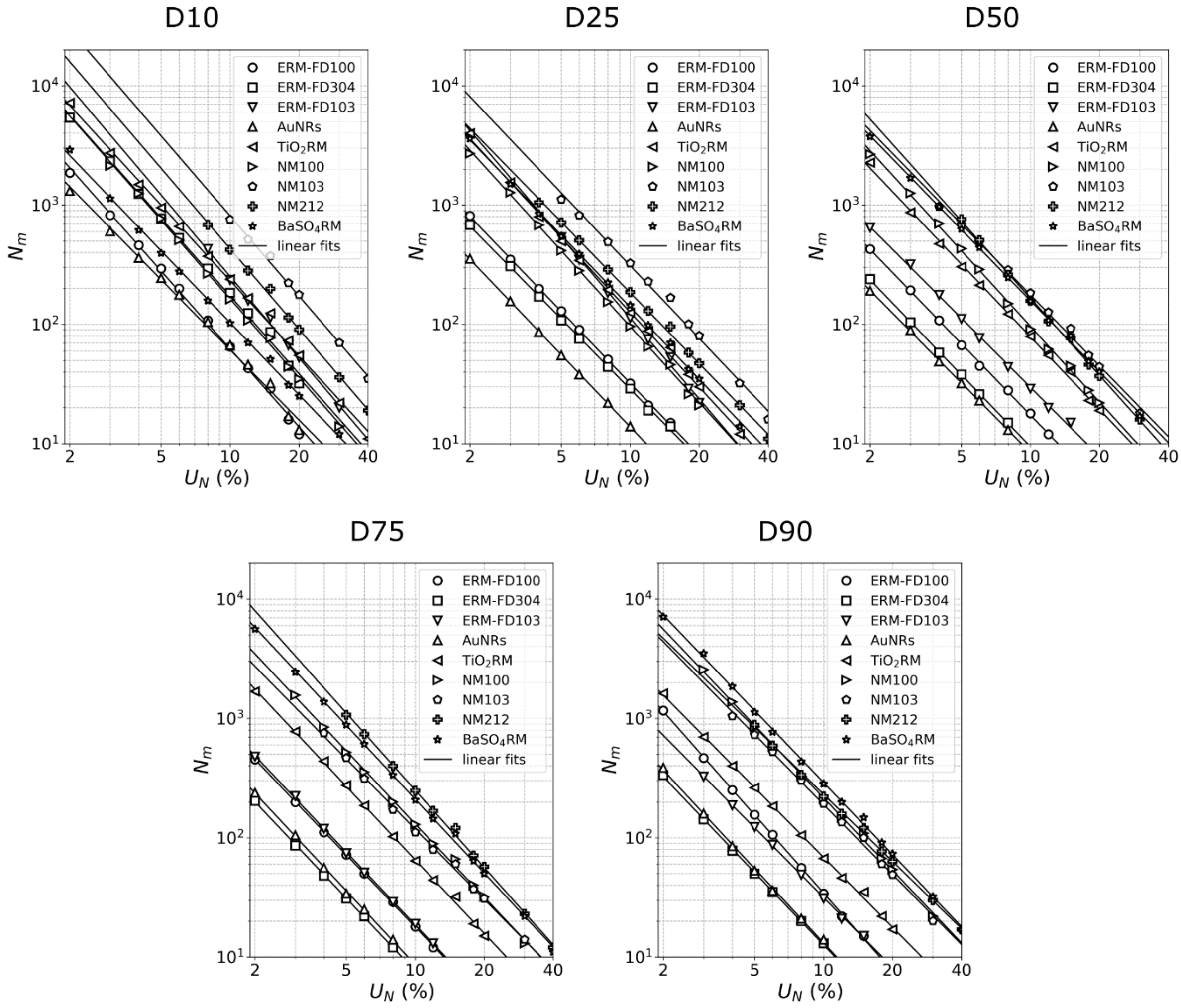

Figure D2: The minimum number of particles as a function of the desired U_N level for the measurement of the percentiles of the Fmax distribution, with linear log-log fits to the data.

ECD

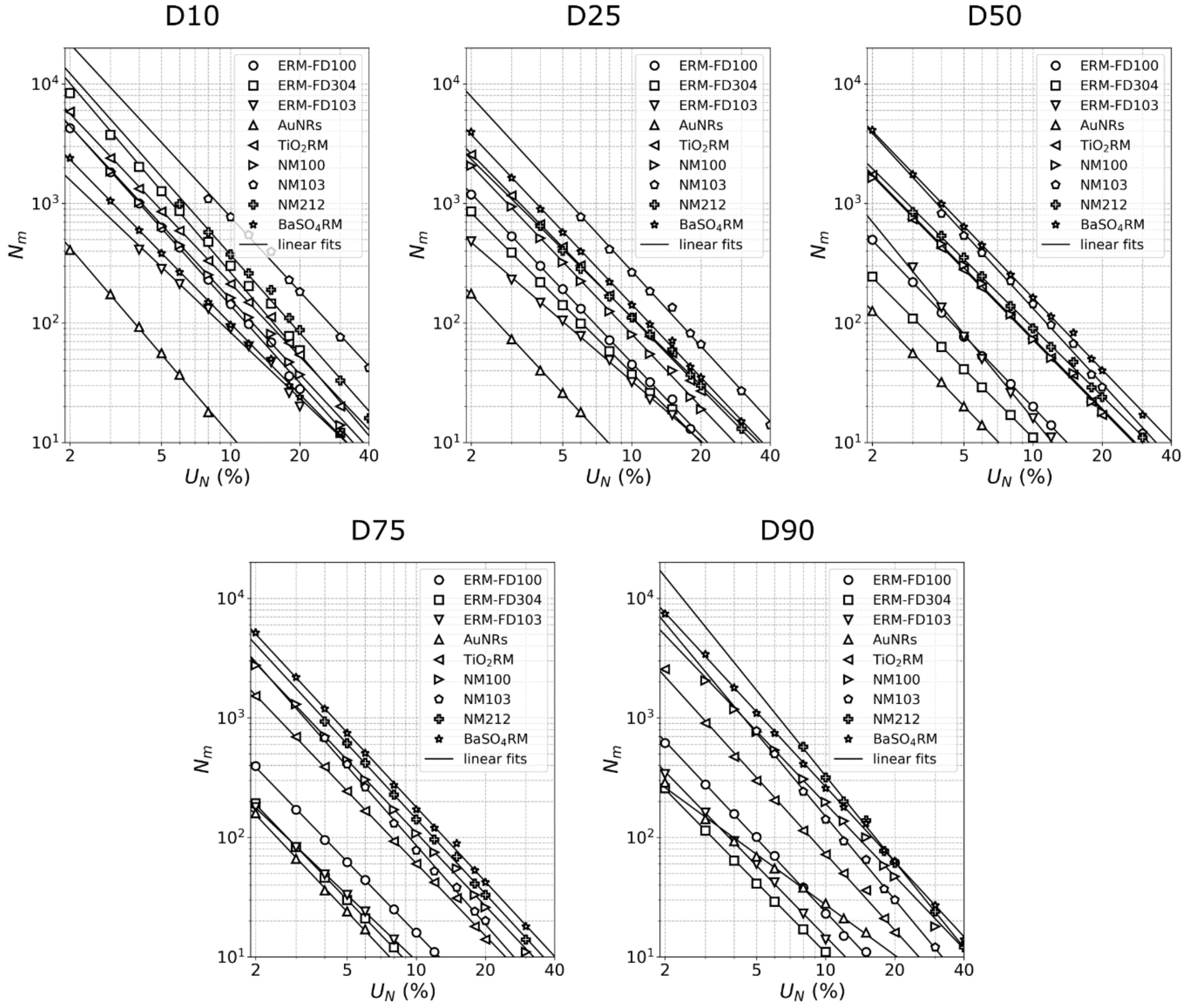

Figure D3: The minimum number of particles as a function of the desired U_N level for the measurement of the percentiles of the ECD distribution, with linear log-log fits to the data.

MICD

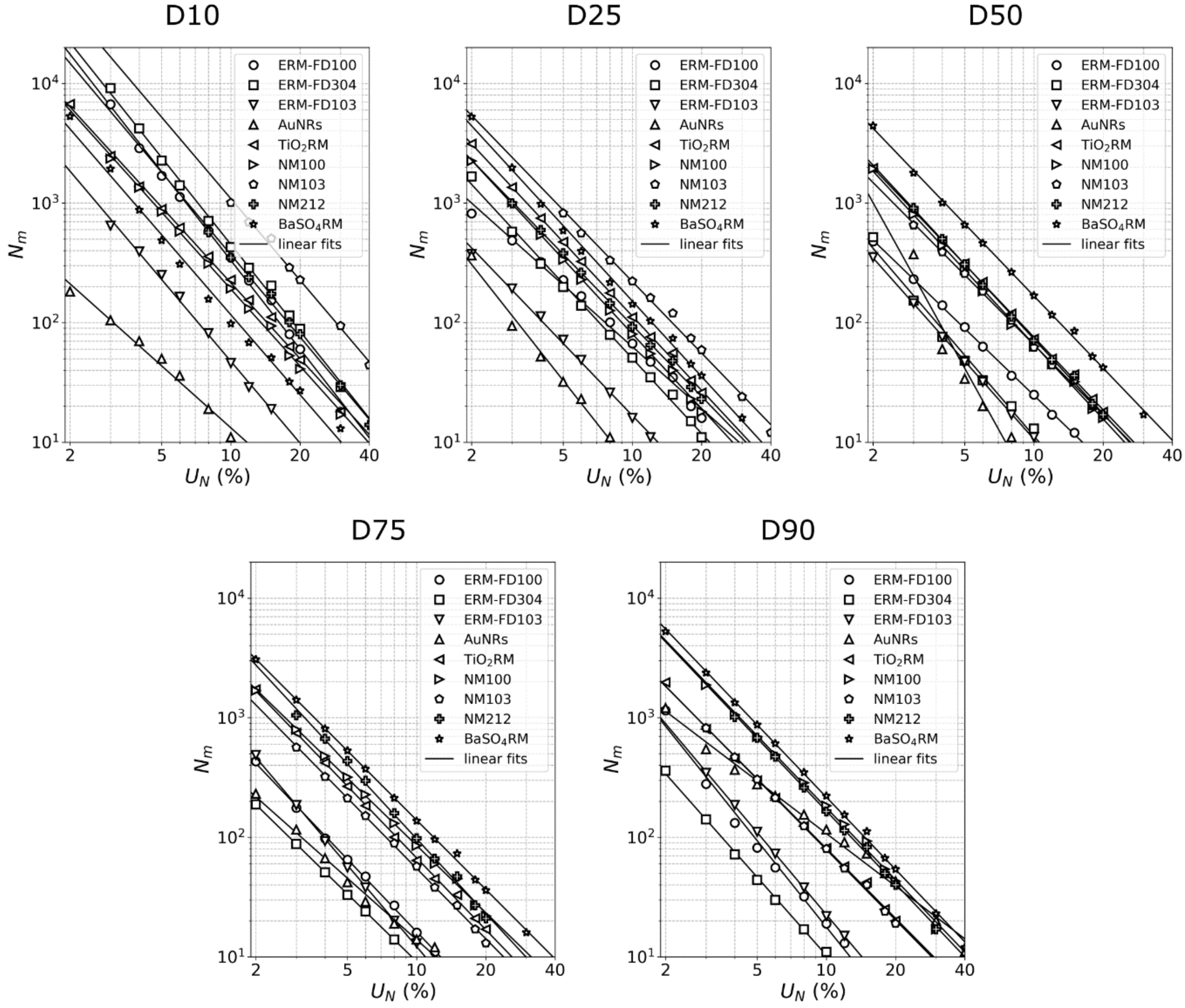

Figure D4: The minimum number of particles as a function of the desired U_N level for the measurement of the percentiles of the MICD distribution, with linear log-log fits to the data.

AR

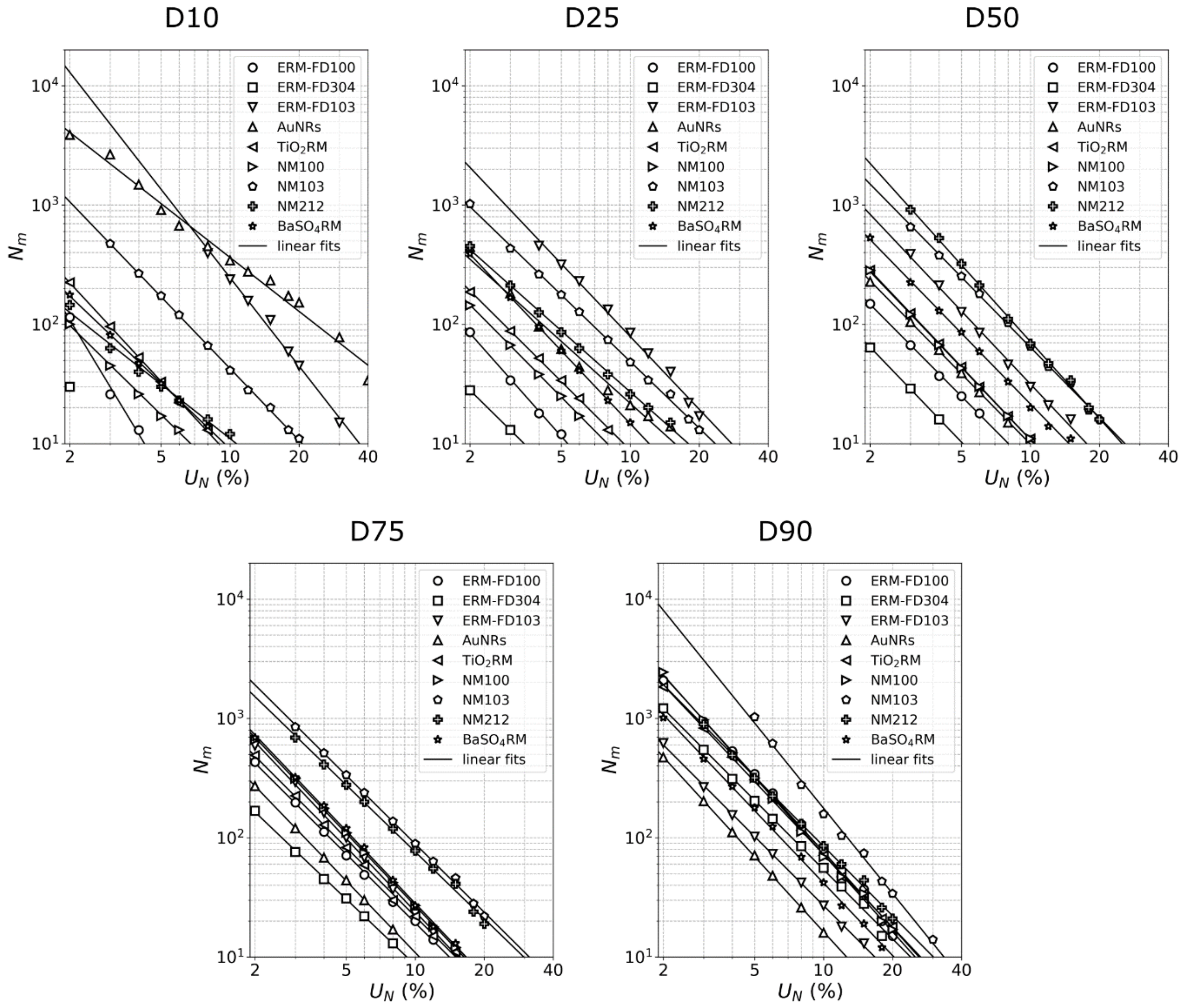

Figure D5: The minimum number of particles as a function of the desired U_N level for the measurement of the percentiles of the Fmin distribution, with linear log-log fits to the data.

Comparison with theory

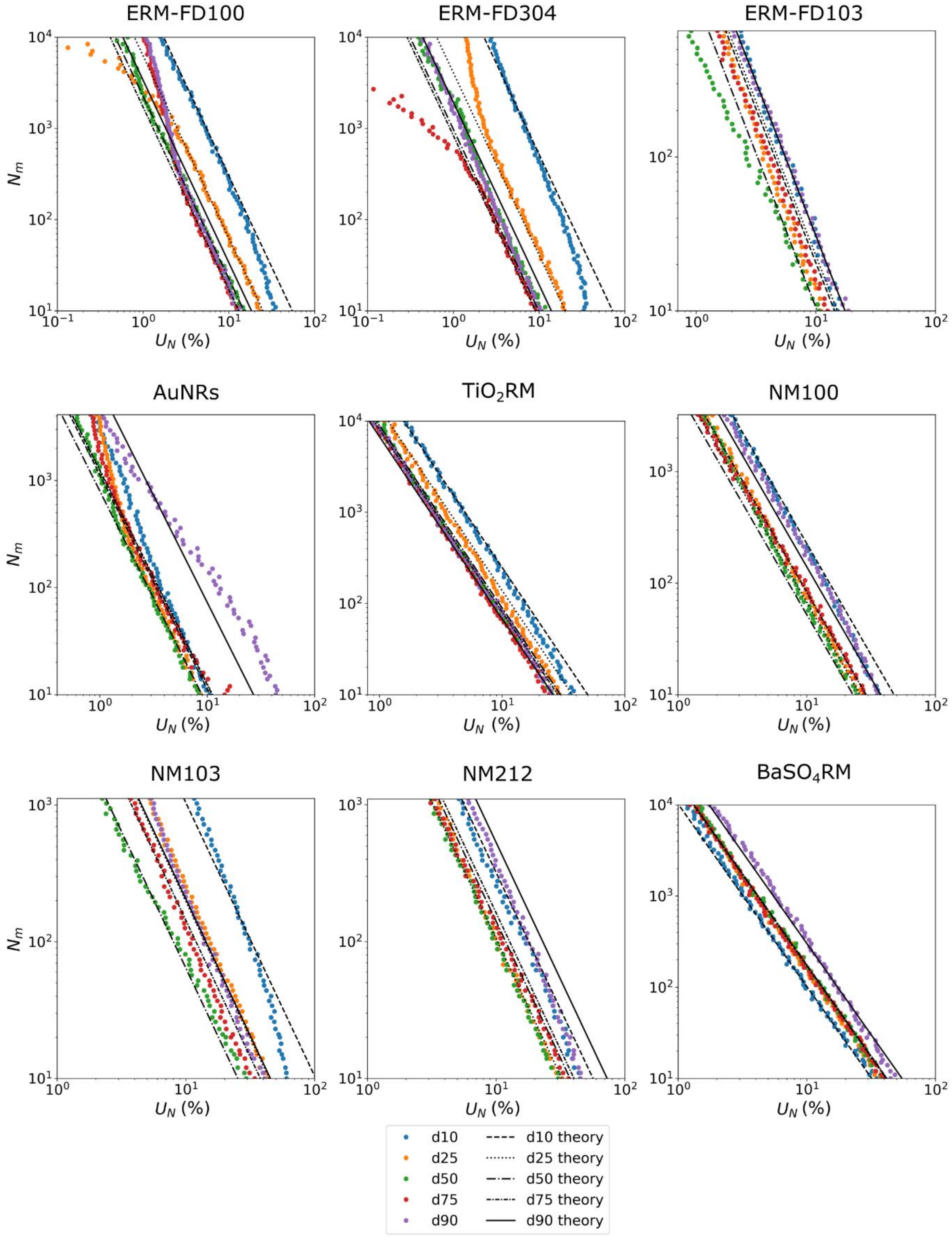

Figure D6: The minimum number of particles as a function of the desired U_N level for measurement of the different percentiles of the Fmin distribution of all considered materials. A comparison of the experimental data with the theoretic derivation by Matsuyama [26] is made.

According to the theoretical derivation by Matsuyama [26], the uncertainty expressed as standard deviation of the percentile D of a measurand x with a discrete probability distribution can be approximated as

$$\sigma_{Dx} = \frac{N_{\mathrm{tot}}\,\Delta x}{n_{\mathrm{D}}}\sqrt{\frac{D(1-D)}{N}},$$

with n_{D} the number of particles in the bin containing the percentile d, N_{tot} the total number particles and Δx the width of the discrete size classes and N the variable particle count. This can be converted to uncertainty with coverage factor $k = 2$ by

$$U_{\mathrm{N}} = 2\,\frac{\sigma_{Dx}}{D}.$$

A good agreement between theory and simulations from a lognormal size distribution were shown by Matsuyama. In Figure D6, we compare the theoretic derivation to the outcome of our calculations of U_{N} vs N_{m} based on real TEM experimental data, with histograms deviating from lognormality. Qualitatively, the data agree well with the theory; both present a linear log-log relationship between U_{N} vs. N_{m} for the majority of the considered range of N for all materials and all percentiles. Deviations can in some cases be observed at high N, either towards lower or higher values of N_{m}. These deviations can probably be attributed to the fact that the measured size values are rounded down by the image analysis software to 0.1 nm. However, the 'real' percentile value of the continuous distribution (which can be represented by the kernel density estimate) might be located in between two rounded values. In such a case, the uncertainty on the percentile will level out towards increasing values of N_{m}. On the other hand when the 'real' percentile value is close to the rounded value, U_{N} will fall back to zero for increasing N since the same rounded value is measured for all samples. Basically, the theoretical limit of uncertainty can not be achieved in reality since other uncertainty contributions are playing a role as well. Deviations at low N are observed as well, especially for D10 percentiles. The D10 percentile is typically the most difficult to estimate due to the fact that it finds itself in the tail of the distribution (with more noise and small contaminating particles), which might explain the deviations from theory at low values of N. Quantitatively, the theory curves also fit quite well to the experimental data. Slight shifts of the data with respect to the curves are observed in some cases. This can be explained by the fact that the position of the curve depends on the representation (i.e. choice of bin width of the data) of the histogram. For data following a well-described distribution, this will not be important but since the experimental data contain some randomness, the choice of bin width might significantly influence the theoretical estimation and thus position of the curve.

E. Estimate of IQR% from limited number of TEM images

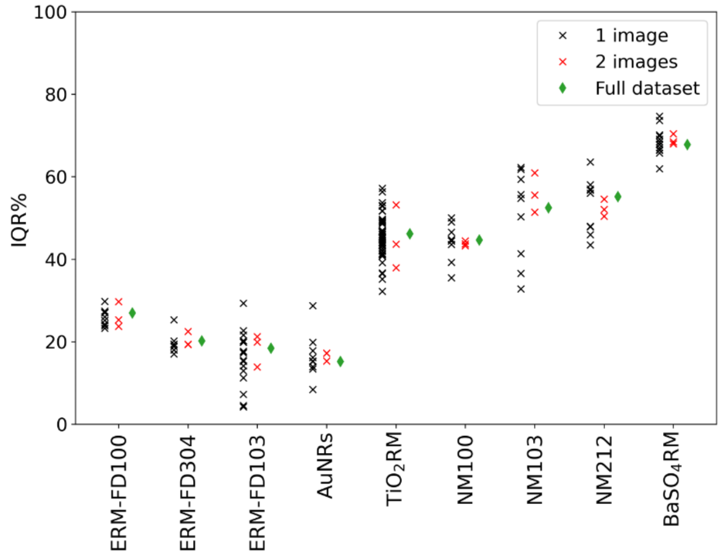

Material	Avg # particles / image
ERM-FD100	1165
ERM-FD304	1715
ERM-FD103	33
AuNRs	412
TiO_2RM	227
NM100	325
NM103	112
NM212	110
$BaSO_4$RM	1168

Figure E1: The graph shows the comparison of IQR% of the Fmin distribution determined from a single or two TEM images (multiple examples) and from the full dataset. The table lists the average number of particles captured in one image for the materials.

F. Robustness against changing image magnification

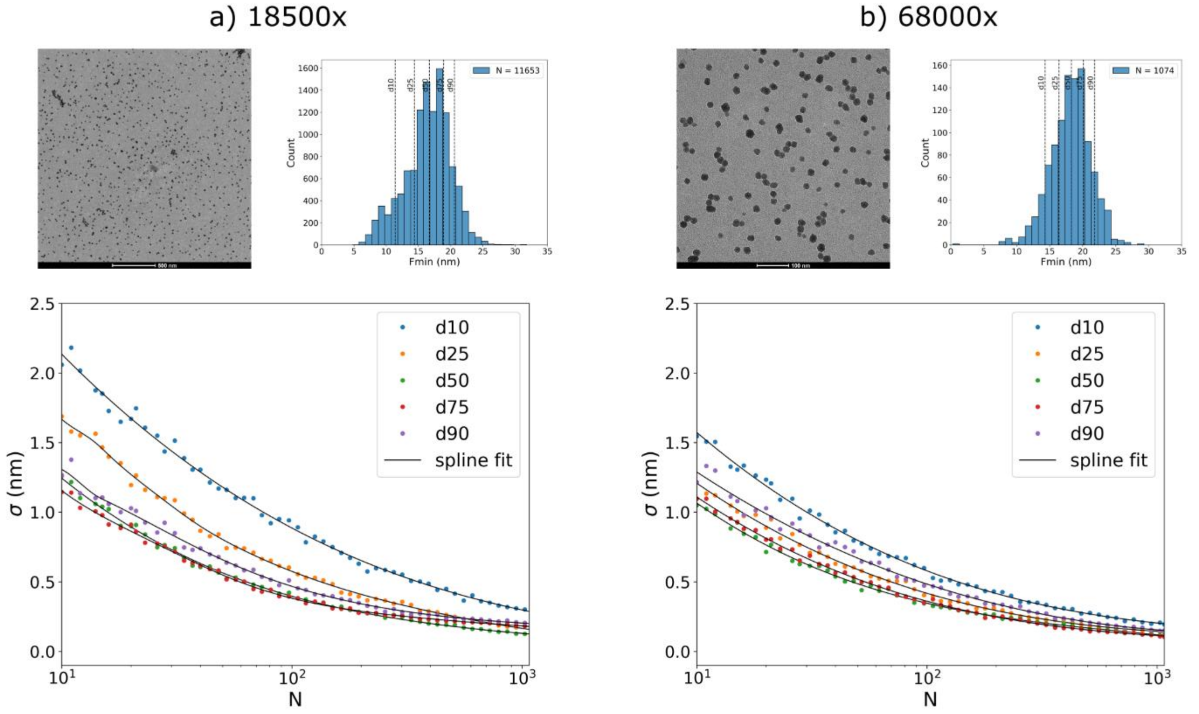

Figure F1: Representative TEM image, Fmin number-based size distribution based on 10 images and variation of the standard deviation of the percentiles of the Fmin distribution as a function of particle number N obtained by sampling 500 times at each value of N, for imaging of ERM-FD100 at a magnification of 18500x (a) and 68000x (b).

Table F1 Summary of the applied imaging and analysis conditions and the descriptive statistics of the Fmin size distributions of ERM-FD100 as analysed in Figure F1.

**Material**	**Magnification**	**ParticleSizer analysis mode**	**N_{tot}**	**D10 (nm)**	**D25 (nm)**	**D50 (nm)**	**D75 (nm)**	**D90 (nm)**	**IQR%**	**U_{cx} (%)[1]**
**ERM-FD100**	18500x [2]	Default	11653	11.4	14.4	16.7	18.9	21.5	27	9
**ERM-FD100**	68000x [3]	Default	1074	14.3	16.4	18.3	20.1	21.8	20	9

[1]Total expanded measurement uncertainty (95 %) for the median (D50) Fmin measurement results obtained from validation studies.
[2]Corresponding to a pixel size of 0.598 nm.
[3]Corresponding to a pixel size of 0.161 nm.

Table F2 Number of particles required to reach the indicated uncertainties on the different percentiles of the Fmin size distributions for the case considered in Figure F1a.

**18500x**	**D10**	**D25**	**D50**	**D75**	**D90**
**$N_m(U_N = 15\ \%)$**	110	29	11	10	10
**$N_m(U_N = 10\ \%)$**	262	63	23	17	19
**$N_m(U_N = 5\ \%)$**	1133	258	91	67	83

Table F3 Number of particles required to reach different uncertainties on the different percentiles of the Fmin size distributions for the case considered in Figure F1b.

**68000x**	**D10**	**D25**	**D50**	**D75**	**D90**
$N_m(U_N = 15\ \%)$	24	10	10	10	10
$N_m(U_N = 10\ \%)$	63	25	14	13	17
$N_m(U_N = 5\ \%)$	271	104	56	54	78

G. Table of N_m values for different measurands and percentiles

Fmin

Table G1 Minimum number of particles required to reach U_N=10 % for the measurement of the indicated percentiles of the Fmin number-based distribution for the selected materials.

Material	**D10**	**D25**	**D50**	**D75**	**D90**
**ERM-FD100**	262	63	23	17	19
**ERM-FD304**	416	47	13	10	11
**ERM-FD103**	31	13	12	17	32
**AuNRs**	11	10	10	16	145
**TiO_2RM**	222	110	75	66	81
**NM-100**	200	78	62	87	191
**NM-103**	-	251	75	124	225
**NM-212**	266	103	99	125	380
**$BaSO_4$RM**	100	153	172	159	334

Table G2 Minimum number of particles required to reach U_N =5 % for the measurement of the indicated percentiles of the Fmin number-based distribution for the selected materials.

Material	**D10**	**D25**	**D50**	**D75**	**D90**
**ERM-FD100**	1133	258	91	67	83
**ERM-FD304**	2140	182	49	34	47
**ERM-FD103**	123	56	38	68	131
**AuNRs**	55	33	30	44	380
**TiO_2RM**	908	478	313	262	305
**NM-100**	859	336	267	320	684
**NM-103**	-	-	255	621	-
**NM-212**	-	484	413	461	-
**$BaSO_4$RM**	422	630	684	608	1433

Fmax

Table G3 Minimum number of particles required to reach U_N =10 % for the measurement of the indicated percentiles of the Fmax number-based distribution for the selected materials.

Material	**D10**	**D25**	**D50**	**D75**	**D90**
**ERM-FD100**	65	32	18	18	34
**ERM-FD304**	184	29	10	10	13
**ERM-FD103**	236	113	29	19	31
**AuNRs**	67	14	10	10	14
**TiO_2RM**	238	125	79	64	67
**NM-100**	162	96	91	129	212
**NM-103**	754	324	183	111	193
**NM-212**	422	186	157	248	222
**$BaSO_4$RM**	102	144	156	207	283

Table G4 Minimum number of particles required to reach U_N =5 % for the measurement of the indicated percentiles of the Fmax number-based distribution for the selected materials.

Material	**D10**	**D25**	**D50**	**D75**	**D90**

**ERM-FD100**	293	129	67	72	156
**ERM-FD304**	769	108	38	31	50
**ERM-FD103**	-	537	111	74	123
**AuNRs**	244	55	32	34	53
**TiO_2RM**	947	498	303	275	262
**NM-100**	769	416	431	519	848
**NM-103**	-	1110	684	465	728
**NM-212**	-	713	759	1072	883
**$BaSO_4$RM**	396	548	630	883	1125

ECD

Table G5 Minimum number of particles required to reach U_N =10 % for the measurement of the indicated percentiles of the ECD number-based distribution for the selected materials.

Material	**D10**	**D25**	**D50**	**D75**	**D90**
**ERM-FD100**	144	45	20	16	23
**ERM-FD304**	301	37	11	10	11
**ERM-FD103**	90	32	16	10	14
**AuNRs**	10	10	10	10	28
**TiO_2RM**	212	111	74	60	72
**NM-100**	162	80	73	108	197
**NM-103**	769	264	144	78	141
**NM-212**	375	111	90	141	316
**$BaSO_4$RM**	96	141	163	171	258

Table G6 Minimum number of particles required to reach U_N =5 % for the measurement of the indicated percentiles of the ECD number-based distribution for the selected materials.

Material	**D10**	**D25**	**D50**	**D75**	**D90**
**ERM-FD100**	630	192	77	62	101
**ERM-FD304**	1266	141	41	30	41
**ERM-FD103**	283	105	77	33	60
**AuNRs**	56	26	20	24	69
**TiO_2RM**	853	434	279	243	297
**NM-100**	647	322	305	440	764
**NM-103**	-	-	537	408	775
**NM-212**	-	402	353	613	-
**$BaSO_4$RM**	383	572	638	743	1095

MICD

Table G7 Minimum number of particles required to reach U_N =10 % for the measurement of the indicated percentiles of the MICD number-based distribution for the selected materials.

Material	**D10**	**D25**	**D50**	**D75**	**D90**
**ERM-FD100**	350	67	25	16	19
**ERM-FD304**	428	51	13	10	11
**ERM-FD103**	46	16	11	13	22
**AuNRs**	11	10	10	14	116
**TiO_2RM**	228	111	73	64	81
**NM-100**	193	80	63	85	184
**NM-103**	1007	222	62	57	80

**NM-212**	350	93	71	98	165
**$BaSO_4$RM**	98	143	168	137	222

Table G8 Minimum number of particles required to reach U_N =5 % for the measurement of the indicated percentiles of the MICD number-based distribution for the selected materials.

Material	**D10**	**D25**	**D50**	**D75**	**D90**
**ERM-FD100**	1692	227	92	65	82
**ERM-FD304**	2262	199	48	33	44
**ERM-FD103**	250	72	47	56	111
**AuNRs**	50	32	34	42	277
**TiO_2RM**	890	474	313	267	299
**NM-100**	848	336	275	318	694
**NM-103**	-	824	257	212	305
**NM-212**	-	380	307	434	679
**$BaSO_4$RM**	488	588	656	530	871

AR

Table G9 Minimum number of particles required to reach U_N =10 % for the measurement of the indicated percentiles of the AR number-based distribution for the selected materials.

Material	**D10**	**D25**	**D50**	**D75**	**D90**
**ERM-FD100**	10	10	10	20	80
**ERM-FD304**	10	10	10	10	56
**ERM-FD103**	238	85	30	23	27
**AuNRs**	343	21	10	10	16
**TiO_2RM**	10	10	11	22	71
**NM-100**	10	10	11	27	69
**NM-103**	41	48	65	89	158
**NM-212**	12	26	69	78	85
**$BaSO_4$RM**	10	15	20	27	42

Table G10 Number of required particles to reach a precision of U_N =5 % for the measurement of the indicated percentiles of the AR number-based distribution for the selected materials.

Material	**D10**	**D25**	**D50**	**D75**	**D90**
**ERM-FD100**	10	12	25	71	343
**ERM-FD304**	10	10	10	31	203
**ERM-FD103**	-	316	127	100	102
**AuNRs**	908	62	39	44	71
**TiO_2RM**	33	34	44	83	305
**NM-100**	17	25	43	110	316
**NM-103**	173	177	253	336	1029
**NM-212**	30	86	322	277	318
**$BaSO_4$RM**	32	60	86	120	178
